# Long-term preservation of Hadean protocrust in Earth’s mantle

**DOI:** 10.1073/pnas.2120241119

**Published:** 2022-04-22

**Authors:** Jonas Tusch, J. Elis Hoffmann, Eric Hasenstab, Mario Fischer-Gödde, Chris S. Marien, Allan H. Wilson, Carsten Münker

**Affiliations:** ^a^Institut für Geologie und Mineralogie, Universität zu Köln, 50674 Köln, Germany;; ^b^Institut für Geologische Wissenschaften, Freie Universität Berlin, 12249 Berlin, Germany;; ^c^School of Geosciences, University of the Witwatersrand, Johannesburg 2050, South Africa

**Keywords:** ^182^W, Hadean, tungsten, geodynamics, early Earth

## Abstract

With plate tectonics operating on Earth, the preservation potential for mantle reservoirs from the Hadean Eon (>4.0 Ga) has been regarded as very small. The quest for such early remnants has been spurred by the observation that many Archean rocks exhibit excesses of ^182^W, the decay product of short-lived ^182^Hf. However, it remains speculative whether Archean ^182^W anomalies and also ^182^W deficits found in many young ocean island basalts (OIBs) mirror primordial Hadean mantle differentiation or merely variable contributions from older meteorite building blocks delivered to the growing Earth. Here, we present a high-precision ^182^W isotope dataset for 3.22- to 3.55-Ga-old rocks from the Kaapvaal Craton, southern Africa. In expanding previous work, our study reveals widespread ^182^W deficits in different rock units from the Kaapvaal Craton and also the discovery of a negative covariation between short-lived ^182^W and long-lived ^176^Hf–^143^Nd–^138^Ce patterns, a trend of global significance. Among different models, these distinct patterns can be best explained by the presence of recycled mafic restites from Hadean protocrust in the ancient mantle beneath the Kaapvaal Craton. Further, the data provide unambiguous evidence for the operation of silicate differentiation processes on Earth during the lifetime of ^182^Hf, that is, the first 60 million y after solar system formation. The striking isotopic similarity between recycled protocrust and the low-^182^W endmember of modern OIBs might also constitute the missing link bridging ^182^W isotope systematics in Archean and young mantle-derived rocks.

## Significance

Due to active plate tectonics, there are no direct rock archives covering the first ca. 500 million y of Earth’s history. Therefore, insights into Hadean geodynamics rely on indirect observations from geochemistry. We present a high-precision ^182^W dataset for rocks from the Kaapvaal Craton, southern Africa, revealing the presence of Hadean protocrustal remnants in Earth’s mantle. This has broad implications for geochemists, geophysicists, and modelers, as it bridges contrasting ^182^W isotope patterns in Archean and modern mantle-derived rocks. The data reveal the origin of seismically and isotopically anomalous domains in the deep mantle and also provide firm evidence for the operation of silicate differentiation processes during the first 60 million y of Earth’s history.

Due to plate tectonic processes, the accessible silicate reservoirs on Earth lost most of their memory of the first ca. 500 Ma of Earth’s history. Hence, our understanding of this time period is largely based on indirect evidence, for example, from geochemical tracers such as short-lived and now extinct nuclide series that all were only active during the first ca. 100 million y after solar system formation (1–3). The detection of terrestrial variability in the relative abundances of short-lived nuclide decay products such as ^182^W, ^142^Nd, and ^129^Xe provides firm evidence that primordial reservoirs were not fully homogenized by mantle dynamics, and played a significant role during the formation of the first continental crust (3–5). The recent discovery of ^182^W, ^142^Nd, and ^129^Xe anomalies in modern mantle-derived rocks (2, 6, 7) demonstrates that ancient reservoirs remain preserved in the deep mantle. Whereas anomalous ^129^Xe and ^142^Nd isotope compositions (ICs) in mantle-derived rocks can primarily be assigned to early planetary outgassing and early silicate differentiation, respectively, the presence of ^182^W isotope anomalies can result from multiple processes. Negative ^182^W anomalies in modern ocean island basalts (OIBs), for instance, were interpreted to result from core–mantle interaction (8, 9). In contrast, Archean rocks mainly exhibit elevated ^182^W compositions. While some interpret prevalent positive ^182^W anomalies in Archean rocks as a result of disproportional accretion (10), others have pointed out that this view may be an oversimplification, as observations from other isotope systematics suggest other processes to be involved. Suggested alternative models invoke metal–silicate segregation, or silicate differentiation in an early magma ocean or during crust–mantle differentiation (11, 12). Others interpreted the elevated ^182^W ICs as resembling a complementary reservoir to the negative ^182^W isotope anomalies observed in modern OIBs, arguing that core–mantle interaction has caused a secular change in the average mantle μ^182^W from ca. +13 to zero (9). Although, in principle, core–mantle interaction may provide a viable explanation for the secular evolution of ^182^W patterns, it remains highly speculative. Hence, other scenarios should also be considered. For instance, isotope anomalies of ^142^Nd in Archean rocks clearly provide evidence for early silicate differentiation having operated during the Hadean, which may potentially have caused complementary ^182^W anomalies (12, 13). However, it has been demonstrated that pristine ^142^Nd–^182^W records are often obscured, either by multistage differentiation processes within the lifetime of ^146^Sm–^142^Nd, after ^182^Hf–^182^W went extinct, or via fluid-controlled second-stage metasomatic overprint of primordial ^182^W patterns (14).

To further evaluate the processes that can account for ^182^W anomalies in Archean rocks, we investigated samples from the eastern Kaapvaal Craton, southern Africa. These lithologies are well suited to search for vestiges of early silicate differentiation, because they were previously shown to display both heterogeneous ^142^Nd and ^182^W compositions (11, 12, 15–17). We performed high-precision ^182^W isotope analyses on a comprehensive suite of 17 samples that range from mantle-derived lithologies of mafic–ultramafic composition to different types of granitoids. By combining ^182^W isotope analysis with high-precision isotope dilution (ID) measurements for high field-strength elements (HFSE), U, and Th, we assessed the sources of the W inventory in mantle-derived rocks. Our samples span an age range from ca. 3.55 Ga to 3.22 Ga, represent the main lithological units of the Ancient Gneiss Complex (AGC), and also comprise the oldest rocks of the Barberton Granite–Greenstone terrain (BGGT). Moreover, most of these samples have previously been analyzed for their ^143^Nd, ^176^Hf, and ^142^Nd compositions (15, 18, 19), and some samples were remeasured here as replicates. Following a previous attempt (20), we combined ^138^La–^138^Ce isotope analyses with ^143^Nd and ^176^Hf systematics to place further constraints on Hadean mantle differentiation processes. To better understand the depletion history of the Kaapvaal mantle, we also investigated ^143^Nd–^176^Hf systematics in ultramafic rocks from the BARB1 and BARB2 drill cores that were drilled as part of the International Continental Drilling Program (ICDP-2009/01, Expedition ID 5047) in the Komati Formation of the BGGT. These samples were previously shown to exhibit highly variable ^143^Nd–^176^Hf compositions (21). In order to assess whether late-accreted material affected ^182^W isotope systematics, we also investigated Ru isotope systematics that were recently introduced as a novel tool to decipher the inventory of late-accreted material in mantle rocks (22). More information about the regional geology and samples is provided in *SI Appendix*.

Measurements of ^182^W ICs followed previously reported protocols (14, 23), slightly modified to yield sufficiently purified solutions for high-precision measurements using a Thermo Fisher Neptune Plus Multicollector ICPMS (MC-ICP-MS) at Cologne. Uncertainties for averages of repeated analysis of sample solutions (95% CI, *n* = 6 to 11) range between ±1.4 and ±5.1 ppm (average ±2.7 ppm). Our intermediate precision is inferred from repeated analyses of in-house rock reference materials, including a 3.27-Ga-old komatiite from the Pilbara Craton (sample 160245, Ruth Well Formation), Western Australia, previously shown to display a ^182^W excess of 6.8 ± 2.3 ppm (23). All in-house rock reference materials were also passed through our separation protocol and measured in every session, yielding 2 SD ≤ ± 2.7 ppm (*SI Appendix*, Fig. S1). More information about the analytical protocol (including ID techniques and IC measurements for Hf, Nd, Ce, and Ru) is provided in *Materials and Methods*.

**Fig. 1.**
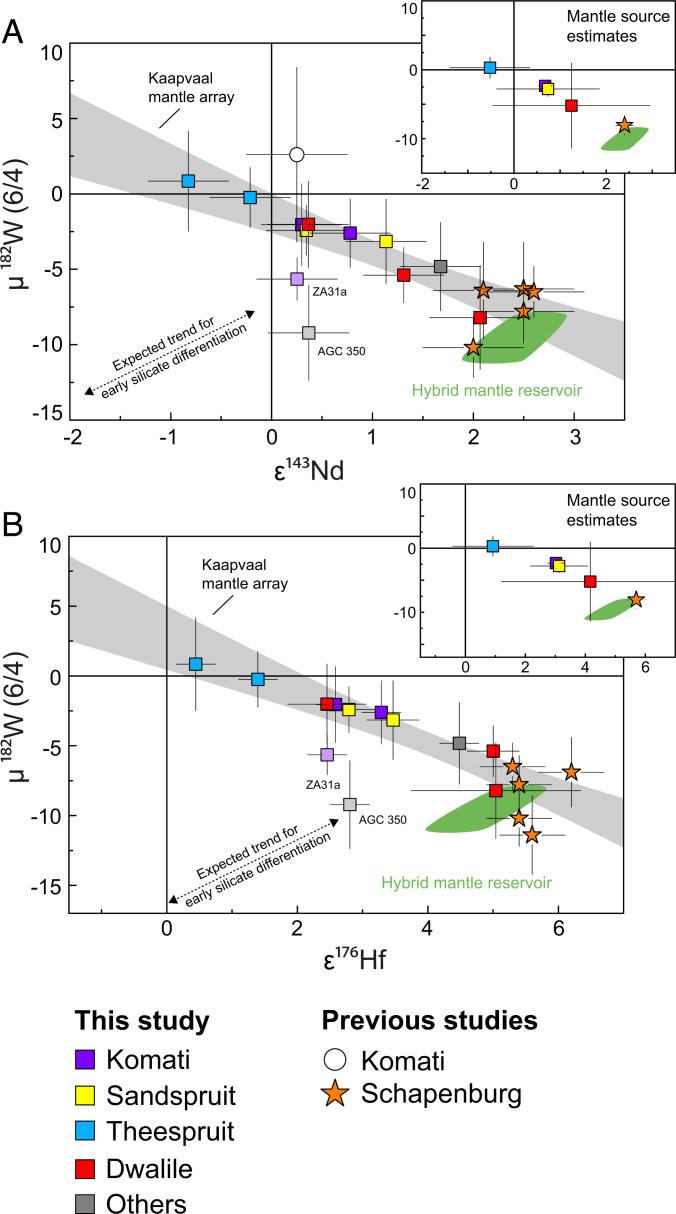
Measured µ^182^W vs. ε^143^Nd_(t)_ (*A*) and µ^182^W vs. ε^176^Hf_(t)_ (*B*) for individual mantle-derived mafic rock samples and mantle source compositions (*Inserts*) from the Kaapvaal Craton, including literature data. The ^182^W IC for sample AGC 350 and ZA31a (pale symbols) were most likely overprinted by metasomatic agents carrying negative ^182^W ICs. The literature data include previously published data for komatiites from the SGR (orange asterisks) (12) and the Komati Formation (open circle) (11, 16). We note that previously published literature data for the Komati Formation only report combined µ^182^W vs. ε^143^Nd_(t)_ data for one single sample (sample BV 02, open circle) (11, 16). The green fields illustrate modeled values of our proposed hybrid reservoir (10 to 20% restites admixed to depleted mantle). The shaded gray field, referred to as Kaapvaal mantle array, is an uncertainty envelope employing the 95% CI in which all mantle-derived samples are expected to fall. Note that the negative covariation displayed by the Kaapvaal mantle array does not follow the expected trend for early silicate differentiation (indicated by dashed line).

Our results for ^182^W isotope analysis are summarized in Dataset S1. Major and trace element compositions as well as ^138^Ce–^142,143^Nd–^176^Hf and Ru ICs are provided in Dataset S2. Irrespective of petrology and provenance (AGC or BGGT), all rock types display ^182^W ICs that range from modern mantle values (μ^182^W = 0) to deficits as low as −9.2 ± 3.2 ppm. While most mantle-derived rocks from the BGGT display μ^182^W values overlapping with the modern mantle value, most mantle-derived rocks from the AGC display resolvable μ^182^W deficits. The distribution and the range of ^182^W ICs in our rock samples from the Kaapvaal Craton are similar to those for ^142^Nd, displaying both negative and modern compositions (15). However, combined ^182^W–^142^Nd data for rocks from the eastern Kaapvaal Craton, including literature data from the Schapenburg Greenstone Remnant (SGR) adjacent to the BGGT (12), display only vague covariation (*SI Appendix*, Fig. S2), even when considering only those samples with pristine W inventory (i.e., canonical W/Th ratios). Notably, our dataset reveals a negative covariation of μ^182^W with initial ε^143^Nd_(t)_ and ε^176^Hf_(t)_ for mantle-derived rocks (Fig. 1) which is not observed for μ^142^Nd. This reveals covariation between ^182^W compositions and long-lived radiogenic nuclides. The observed covariation for our samples is further strengthened by literature data for komatiites from the SGR adjacent to the BGGT (12) and the Komati Formation from the BGGT (11, 16). The absence of similar covariations in other Archean lithostratigraphic successions can be explained either by initial igneous processes that decoupled ^143^Nd–^176^Hf systematics during source overprint (24) or by the disturbance of pristine ^182^W patterns by metasomatic agents during late-stage metamorphism (13, 14).

**Fig. 2.**
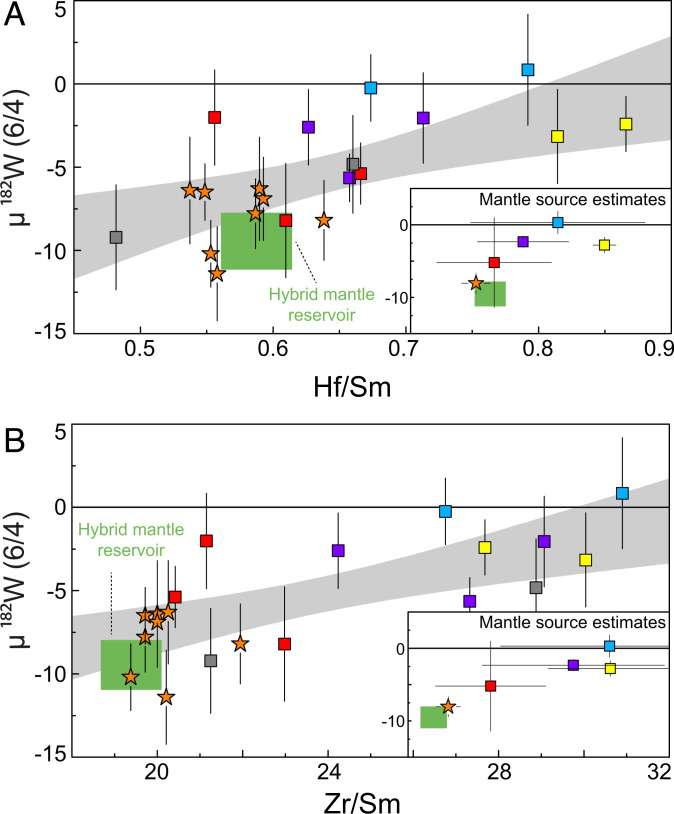
Plot of µ^182^W vs. Hf/Sm (*A*) and Zr/Sm (*B*) for individual mantle-derived mafic rock samples and mantle source compositions (*Inserts*). Symbols are the same as in Fig. 1. Data for komatiites from the SGR were taken from the literature (12). The combined data indicate a systematic covariation between ^182^W IC and Hf/Sm and Zr/Sm ratios with one endmember defined by the SGR komatiites. The negative µ^182^W anomalies and low Hf/Sm and low Zr/Sm ratios prominent in the SGR komatiites can be attributed to the presence of 10 to 20% garnet-rich restites within a hybrid source that underwent 20 to 30% batch melting (green box). The gray shaded array refers to the 95% CI in which of all samples are expected to fall.

A previous study (14) has shown that pristine ^182^W isotope signatures can be modified during fluid-mediated second-stage enrichment of W. One valuable tool capable of screening disturbed elemental W budgets in mantle-derived rocks is the W/Th ratio, which displays a canonical range in pristine magmatic systems (0.09 to 0.24) (25). The majority of samples analyzed in this study display elevated W/Th ratios. These reflect fluid-mediated redistribution of W during metasomatism, as also evident from negative correlations with Ce/Pb (*SI Appendix*, Fig. S3). However, although only three mantle-derived rocks studied here reveal undisturbed elemental W systematics (W/Th ≤ 0.24), the samples still display ^182^W covariations with initial ε^143^Nd_(t)_ and ε^176^Hf_(t)_ values. These observations indicate that the W redistribution did not significantly change the ^182^W composition of these samples and was only of localized character, in contrast to previous studies from other Archean cratons (14, 23). Moreover, covariations with ^182^W compositions are also observed for incompatible trace element ratios classically interpreted as immobile, in particular, Hf/Sm and Zr/Sm (Fig. 2 *A* and *B*). In addition, broadly coupled variations with Zr contents (*SI Appendix*, Fig. S4) demonstrate that Hf and rare-earth elements (REE) were largely immobile during metamorphism. Consequently, a metasomatic origin of the observed covariations between ^182^W and other radiogenic isotopes can be ruled out. As the elements involved display vastly different mobilities at metamorphic conditions, it would be expected that alteration would obscure the observed covariations rather than forming them.

**Fig. 3.**
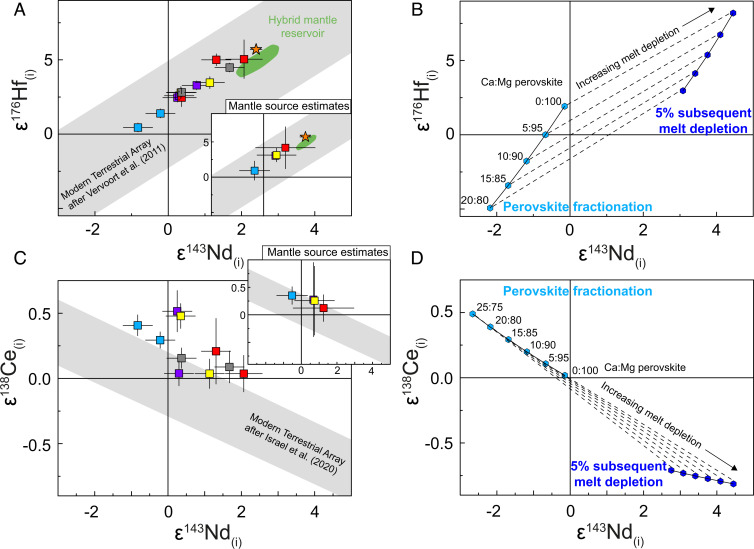
Plot of ɛ^176^Hf_(t)_ vs. ɛ^143^Nd_(t)_ (*A* and *B*) and ɛ^143^Nd_(t)_ vs. ɛ^138^Ce_(t)_ (*C* and *D*) for mantle-derived mafic–ultramafic rocks from the Kaapvaal Craton analyzed in this study (red symbols in *A* and *C*) and for modeled compositions involving perovskite fractionation (blues symbols in *B* and *D*). Estimated mantle source compositions are shown as *Inserts* in *A* and *C*. Symbols are the same as in Fig. 1. The green fields illustrate modeled values of our proposed hybrid mantle reservoir (10 to 20% restites admixed to depleted mantle). Blue symbols in *B* and *D* illustrate our modeling results for mantle reservoirs (at 3.55 Ga) that underwent perovskite segregation (pale blue symbols) and subsequent melt depletion (using an internally consistent set of partition coefficient; see *Materials and Methods*) in the garnet stability field (dark blue symbols) illustrating that ^143^Nd–^176^Hf systematics are not diagnostic features to identify perovskite fractionation in an early magma ocean. The gray bands in *A* and *C* show the modern terrestrial array for MORBs and OIBs (εHf = 1.55 × εNd + 1.21 and εCe = −0.14 × εNd + 0.05) (36, 38).

Most of the samples analyzed in this study also reveal strong correlations between their initial values of long-lived radiogenic isotopes like ɛ^143^Nd_(t)_, ɛ^176^Hf_(t)_, and ɛ^138^Ce_(t)_ (Fig. 3 *A* and *B*). Only two samples (AGC 38 and ZA-38) display disturbed initial ɛ^138^Ce_(t)_ values but still preserve pristine ε^143^Nd_(t)_ and ε^176^Hf_(t)_ systematics. Therefore, initial ɛ^138^Ce_(t)_ values for these samples are excluded from further interpretations and are not shown in Fig. 3*C*. In this regard, combined ^143^Nd–^176^Hf–^138^Ce systematics serve as a valuable tool to clarify why two mantle-derived rocks (AGC 350 and ZA-31a; pale symbols in Fig. 1) deviate slightly from the µ^182^W vs. ε^143^Nd_(t)_ and ε^176^Hf_(t)_ trends. The deviation of these samples toward more negative ^182^W compositions most likely reflects that, in some rare cases, metasomatic agents redistributed W between different reservoirs. As the observed covariations of µ^182^W with ε^143^Nd_(t)_ and ε^176^Hf_(t)_ are defined by mafic–ultramafic volcanic rocks, it is obvious that the observed trend reflects mixing between different mantle source reservoirs. Taking into consideration that postemplacement processes such as hydrothermal alteration or metamorphism may have affected the isotope and trace element systematics of individual samples, we also estimated source compositions for the Komati, Sandspruit, and Theespruit Formations as well as for the Dwalile and SGR (for details, see *Materials and Methods* and Dataset S2). The respective source compositions are shown together with individual sample data as Figs. 1–3, *Inserts*. One mantle endmember exhibits no resolvable ^182^W isotope anomalies at near-chondritic initial ε^143^Nd_(t)_ and ε^176^Hf_(t)_ values, most likely representing near-primitive mantle. The other endmember is best characterized by komatiites from the SGR that exhibit the largest ^182^W isotope deficits extending to −11.4 ppm and strongly elevated initial ε^143^Nd_(t)_ and ε^176^Hf_(t)_ values of up to +2.6 and +6.2, respectively (12). It is surprising that felsic samples from the Kaapvaal Craton plot on the same trend as mafic samples, suggesting short residence times between emplacement of the mafic protolith and formation of felsic orthogneisses (open symbols in *SI Appendix*, Fig. S5).

Our high-precision Ru isotope measurements for two komatiites from the Dwalile Greenstone Remnant (AGC 83 and AGC 86) reveal that the Archean mantle in the Kaapvaal Craton already had, by this stage, a modern mantle–like Ru IC and does not show coupled ^100^Ru–^102^Ru excesses that were recently reported for 3.8- to 3.7-Ga-old Archean rocks from SW Greenland (22). The distinct Ru isotope signature inferred for the SW Greenland rocks was interpreted to reflect a mantle source that did not receive the full complement of late-accreted material (22). In contrast, the modern mantle–like Ru IC of the Dwalile komatiites indicates that the Kaapvaal mantle source by 3.46 Ga had already completely equilibrated with late-accreted material (*SI Appendix*, Figs. S6 and S7).

In the following discussion, we will largely focus on the origin of the low ^182^W endmember. As we will show, the low ^182^W endmember may provide insights into the secular evolution of ^182^W in Earth’s mantle. In particular, we evaluate the concept that present-day mantle plumes with their characteristic ^182^W deficit may be modern analogs of the low-^182^W endmember from the Kaapvaal Craton. So far, ^182^W deficits have been explained as the consequence of several processes. These include 1) equilibration of the mantle source with anomalously large amounts of late-accreted material (late-accretion hypothesis), 2) core–mantle interaction (8, 9, 26), or 3) early fractionation of Hf from W by silicate crystal–liquid fractionation, for example, in an early magma ocean (11).

The late-accretion hypothesis has been postulated to explain the relative and absolute abundances of highly siderophile elements (HSE) in the bulk silicate Earth (BSE) by the addition of about 0.5% of chondritic material after core formation (27, 28). Late accretion would have affected not only the HSE budget of the BSE but also its ^182^W IC (3). Accordingly, some portions of the Archean mantle could have remained in disequilibrium (29), and mantle domains that did not fully equilibrate with late-accretionary components would be characterized by positive ^182^W isotope anomalies and HSE abundances that are lower than the modern BSE. Consequently, negative ^182^W isotope anomalies would imply excesses of late-accreted components that should also be reflected in unusually high HSE contents. However, absolute HSE abundances in the mantle source of the SGR-like endmember with its large ^182^W deficit were estimated to only amount to ca. 30% of those in the present-day BSE (12, 30). The depleted mantle source of the SGR komatiites may provide an explanation here, where the platinum group elements (PGE) depletion may indicate sulfur-undersaturated melting conditions (30). At such conditions, mainly iridium-like platinum group elements (Os, Ir, Ru), that are hosted by refractory platinum group minerals (PGM) remain, to large degrees, in the source (31). This still leaves a realistic possibility that the ^182^W isotope deficits reflect an excess of late-accreted components. However, our constraints from modern mantle–like Ru isotopes clearly demonstrate that the ambient mantle in the Kaapvaal Craton did not receive unusual amounts of late-accreted components (*SI Appendix*, Fig. S7). Moreover, a mantle source that experienced full sulfur exhaustion would likely be extremely depleted in W, making a direct contribution to the ^182^W inventory of the Kaapvaal rocks unlikely.

An alternative explanation for negative ^182^W isotope anomalies in Archean rocks, like those from the Kaapvaal Craton, may be offered by recent studies on OIBs. It has been proposed that prevalent negative ^182^W isotope anomalies in modern, plume-derived OIBs result from chemical and isotopic equilibration between their mantle sources and the outer core without affecting HSE abundances (8, 9, 26). For the same reasoning as outlined above, we regard such a scenario as unlikely. A mantle source that experienced full sulfur exhaustion by large degrees of melt extraction would likely be extremely depleted in W, an incompatible lithophile element, making a direct contribution to the ^182^W inventory of the Kaapvaal rocks most unlikely. Moreover, the modern mantle–like Ru isotope signatures in our samples do not support isotopic equilibration between mantle and core materials. Based on previous constraints on the Ru IC of the prelate veneer mantle (22), the Ru in the core would most likely be characterized by a ^100^Ru excess.

Notably, selective addition of W via core–mantle interaction is not the only explanation for the negative ^182^W anomalies in modern OIBs. Noble gas studies on modern mantle-derived rocks rather suggest that the source reservoirs must have differentiated from the convecting mantle very early and prior to 4.45 Ga (5, 32, 33). The concurrent ^182^W isotope anomalies in the modern mantle may therefore also reflect in situ decay of ^182^Hf (i.e., during the first ca. 60 Ma after solar system formation). The presence of such ancient mantle reservoirs and the role of mantle plumes in the past, in particular their contribution to the secular evolution of the ^182^W IC in the BSE, has so far been only poorly constrained. Notably, it is argued that Archean mafic–ultramafic sequences, like those in the BGGT, also originated from a mantle plume setting (34). In this regard, mantle-derived rocks from the Kaapvaal Craton may have preserved vestiges of ancient mantle heterogeneities, similar to young OIBs.

It has been postulated that recycling of crustal material is responsible for the geochemical and isotopic variability in modern plume-related OIBs (35). In the case of the Kaapvaal Craton, however, direct recycling of ancient protocrust formed during the first ca. 60 Ma appears unlikely, because, in this case, the negative ^182^W and ^142^Nd anomalies should be coupled with unradiogenic ^143^Nd and ^176^Hf compositions. Coupled deficits of ^182^W and ^142^Nd with depleted Hf–Nd isotope patterns led previous studies (12, 17) to conclude that the komatiites from the SGR were derived from a mantle domain that was enriched very early (ca. 30 Ma after solar system formation) in highly incompatible elements as a result of fractionating a Mg- and Ca-perovskite mineral assemblage in an early magma ocean. When originally proposed (12), this conclusion was mainly based on apparently decoupled initial ε^143^Nd_(t)_ and ε^176^Hf_(t)_ compositions of BGGT rocks (16, 21), with particular application to rocks from the Komati Formation (black symbols in *SI Appendix*, Fig. S12). However, more recent work reinvestigated mafic–ultramafic samples from the BGGT (Komati, Sandspruit, and Theespruit Formations) (18) and AGC (Dwalile Greenstone Remnant) (19) by employing more sophisticated sample dissolution protocols, yielding considerably less scatter (red symbols in *SI Appendix*, Fig. S12). By extending this more recent work, we reinvestigated initial ɛ^143^Nd_(t)_ and ɛ^176^Hf_(t)_ compositions in ultramafic rocks from the BARB1 and BARB2 cores that were drilled into the Komati Formation and previously reported to exhibit strongly decoupled ^143^Nd–^176^Hf systematics (21). In fact, together with komatiites from the SGR (12), the new data now fall on a trend closely resembling the modern mantle array (36), indicating that the terrestrial Hf–Nd mantle array had already been established on early Earth (37) (Fig. 3*A* and *SI Appendix*, Fig. S12). In line with our results for ^143^Nd–^176^Hf, initial ɛ^138^Ce and ɛ^143^Nd systematics also closely fall on the modern terrestrial array (38). On the basis of available literature data, we cannot rule out that other mafic units in the BGGT (e.g., Weltevreden; blue field in *SI Appendix*, Fig. S12) do indeed preserve extreme decoupling in their ɛ^143^Nd and ɛ^176^Hf systematics and anomalous ɛ^138^Ce. However, a recent study on the 3.33-Ga Commondale komatiites from the Kaapvaal Craton demonstrates that decoupled ^143^Nd–^176^Hf patterns are not unique to magma ocean relics but can also be generated via hybrid melting of depleted mantle and garnet pyroxenites in the stability field of garnet (20, 39).

On the basis of more rigorous modeling, employing updated sets of partition coefficients (40, 41), and by adopting a previous model for SGR komatiites (12), we therefore reevaluated the control of perovskite segregation and subsequent mantle depletion on the ^143^Nd–^176^Hf–^138^Ce-^142^Nd isotope inventory (details are provided in *Materials and Methods*, and calculations are provided in Dataset S3). Herein, a primitive mantle undergoes removal of 10% of the perovskite cumulate (Ca:Mg-perovskite 5:95) at 4.537 Ga before it evolves until 4.027 Ga. Subsequently, this reservoir undergoes melt depletion at 4.027 Ga before it melts at 3.55 Ga to produce the SGR komatiites. In Fig. 3, we show the evolution of such a mantle source composition recalculated to 3.55 Ga in ^143^Nd–^176^Hf (Fig. 3*B*) and ^143^Nd–^138^Ce space (Fig. 3*D*) as a function of variable Ca:Mg-perovskite proportions (Ca:Mg-perovskite from 20:80 to 0:100). Considering that melt depletion at 4.027 Ga took place in the garnet stability field, the modeled results for ^143^Nd–^176^Hf (Fig. 3) are in reasonable agreement with the SGR komatiites. However, fractionating a Ca:Mg-perovskite assemblage of 5:95 as previously suggested (12) does not lead to suprachondritic Lu/Hf and to decoupling of initial ε^143^Nd and ε^176^Hf systematics, once recalculated to 3.55 Ga. Rather, the Hf–Nd composition of the modeled mantle after perovskite segregation is near-chondritic at 3.55 Ga. It is the second-stage differentiation step in the garnet stability field at 4.027 Ga that generates the ε^143^Nd–ε^176^Hf systematics observed in SGR komatiites. This finding, and the fact that mantle-derived rocks from the Kaapvaal Craton follow the modern-day terrestrial Hf–Nd array, suggests that this array had already started to form in the Archean, as a consequence of deep-mantle melting and an increased role of residual garnet together with early crustal recycling (37, 42).

The negligible impact of fractionating 10% of the perovskite cumulate on magma compositions becomes even more obvious when the incompatible trace element budgets of both reservoirs are plotted relative to primitive mantle (*SI Appendix*, Fig. S8). More importantly, the relative proportions of Ca- and Mg-perovskite in the cumulate exert a strong influence on ^143^Nd–^176^Hf systematics (24). It becomes apparent that ^176^Hf–^143^Nd decoupling strongly depends on the proportion of Ca-perovskite crystallizing with Mg-perovskite. However, coprecipitation of Ca- and Mg-perovskite during the first 10% of the fractional crystallization in a magma ocean remains an open issue, as it is highly unlikely that Ca-perovskite appears as the first liquidus phase at lower-mantle conditions (43–45). On the basis of these combined arguments, we conclude that ^143^Nd–^176^Hf isotope systematics in the Kaapvaal rocks are nondiagnostic for fractionation of perovskite cumulates. Most importantly, the perovskite model has difficulties explaining why ^142^Nd compositions in mantle-derived rocks from the Kaapvaal Craton do not correlate with ^143^Nd–^176^Hf compositions.

**Fig. 4.**
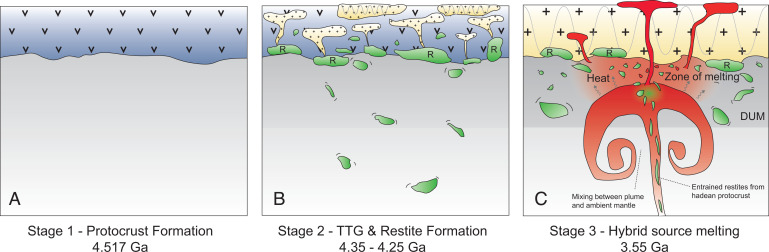
Preferred geodynamic model for the origin of crustal and mantle-derived rocks from the Kaapvaal Craton. (*A*) Formation of a mafic protocrust by ca. 50 Ma after solar system formation. (*B*) Formation of TTG-like batholiths (orange) and residual garnet-rich restites (green, labeled “R”) after partial protocrustal anatexis between ca. 4.35 and 4.25 Ga. (*C*) Recycling of lower-crustal restites and plume-initiated volcanism lead to melting of hybrid sources that involved delaminated restites, depleted and primitive mantle supplied by the ascending plume. Shades of gray visualize depleted upper mantle (DUM) and lower mantle (light gray). Note that delaminated restites may either be probed by deep-rooted mantle plumes from lower-mantle regions or be assimilated in the upper mantle by ascending plumes.

Based on these considerations, a two-stage process is clearly required, where the negative ^182^W and ^142^Nd anomalies formed early and the radiogenic ^143^Nd and ^176^Hf compositions were established after the short-lived systems became extinct. Our preferred geodynamic model is illustrated in Fig. 4, a detailed description of which is given in *Materials and Methods*, and all model parameters and calculations are provided in Dataset S3. Our model is inspired by previous studies on the mechanisms of formation of early continental crust (46–48). Accordingly, after formation of a mafic protocrust (Fig. 4*A*), intracrustal fractionation led to the formation of a felsic, TTG-like crust and mafic lower-crustal restites that were recycled into the mantle due to their high densities. Here, they mechanically and chemically interacted with mantle peridotites, producing hybrid mantle reservoirs (Fig. 4*B*). These hybrid mantle reservoirs were probed either by deep-rooted mantle plumes from lower-mantle regions (49) or by assimilation by the upper mantle during plume upwelling (50) (Fig. 4*C*). Melting of such hybrid reservoirs in conjunction with near-primitive mantle may thus account for the compositional trend between long-lived decay systems and ^182^W, as observed for mafic rocks from the Kaapvaal Craton. In this scenario, the near-primitive mantle endmember is characterized by the Barberton komatiites, and the hybrid mantle endmember is characterized by the SGR komatiites. As demonstrated below, such mixing relationships provide a viable explanation for the negative covariation between short- and long-lived radiogenic systems. Moreover, our model can also explain the incompatible trace element systematics in our samples and the SGR komatiites (*SI Appendix*, Figs. S4 and S13).

Following constraints from phase equilibrium and trace element modeling, melting of Archean TTG suites from mafic protocrust leaves behind residual assemblages of amphibolitic, garnet-amphibolitic, or garnet-pyroxenitic composition (51). Fig. 4*A* illustrates that, during stage 1, mafic protocrust that formed 50 Ma after solar system formation developed strongly unradiogenic ICs, in particular for the short-lived decay products ^182^W and ^142^Nd. Subsequent TTG melting (stage 2 in Fig. 4*A*) leaves behind garnet-rich restites (52), and, depending on the timing of this second event, the residual restites will develop toward markedly different ^142^Nd ICs with time. In contrast, the ^182^W IC will be insensitive to the timing of TTG extraction, because ^182^Hf became extinct shortly after formation of the protocrust. Evidence for the presence of such ancient TTG precursors in the Kaapvaal Craton comes from Hf isotope-in-zircon data (53–55) and from rare Hadean detrital zircons in the ca. 3.3-Ga Fig Tree Formation (56) that suggest formation of a felsic protocrust already by the Eoarchean or Late Hadean. Moreover, a recent study investigated ^182^W isotope systematics in diamictites from the Kaapvaal Craton and revealed that the exposed upper continental crust at that time must have had negative ^182^W compositions (57). Due to the longer half-lives of their parent nuclides, ^143^Nd and ^176^Hf ICs in the restites integrate a larger time span and develop much less heterogeneity with time than ^142^Nd, which can only be formed over a smaller time interval until ^146^Sm becomes extinct. These considerations explain why ^142^Nd signatures became quite variable, depending on the time of TTG extraction, unlike long-lived Hf–Nd compositions that persistently developed toward slightly radiogenic values over time.

**Fig. 5.**
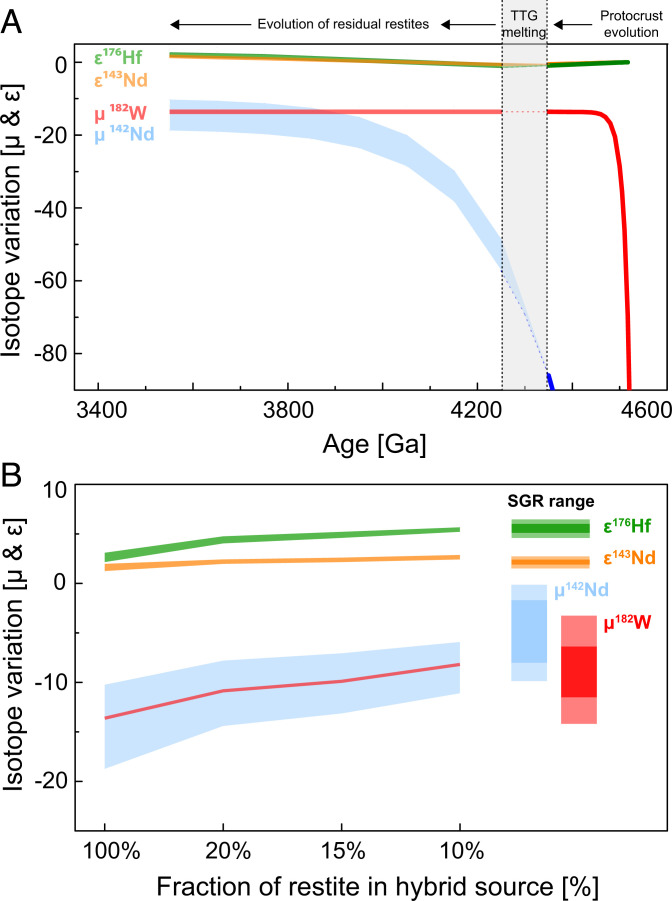
Isotope evolution graphs for the proposed geodynamic model involving mantle recycling of lower-crustal restites. (*A*) During stage 1, mafic protocrust formed ca. 50 Ma after solar system formation developed strongly unradiogenic ICs, in particular for ^182^W and ^142^Nd. Stage 2 marks restite formation during TTG extraction from mafic protocrust. The gray bar illustrates the time interval (4.35 Ga to 4.25 Ga) over which TTG extraction affects the ICs of residual restites. Depending on the exact timing of TTG extraction, the restites develop to markedly different ^142^Nd IC with time (blue field). In contrast, ^182^W is insensitive to the timing of TTG extraction, because ^182^Hf went extinct shortly after formation of the mafic protocrust. Due to their longer half-lives, the effects on long-lived radionuclides are rather negligible. (*B*) Mixing calculations illustrating the IC of the proposed hybrid reservoirs as a function of delaminated restites mixed into depleted mantle. Approximately 10 to 20% of admixed restite to depleted mantle reproduces the ICs found in the SGR endmember. This hybrid source mixed with primitive mantle material supplied by ascending mantle plumes as reflected in the Kaapvaal mantle array for ^182^W and long-lived radiogenic nuclides (Fig. 1).

It becomes apparent from Fig. 5*A* that lower-crustal restites from ancient protocrust can explain why the ^143^Nd and ^176^Hf ICs are tightly correlated with ^182^W but not with ^142^Nd. Recycling of such restites into the mantle formed a hybrid source that is best approximated by compositions of Schapenburg komatiites. We found that 10 to 20% of restites admixed to depleted mantle already reproduce the radiogenic ICs found in the SGR endmember (Fig. 5*B*). Once this hybrid mantle source is mixed with primitive material supplied by ascending mantle plumes, it can account for the systematic coupling between initial ε^143^Nd_(t)_–ε^176^Hf_(t)_ and ε^143^Nd_(t)_–ε^138^Ce_(t)_ (Fig. 3) and also for the opposite variations of ^182^W (Fig. 1). Notably, our proposed model can well reproduce the incompatible trace element compositions and reconcile distinct trace element features that are diagnostic for the SGR komatiites. Exact modeling of ^138^La–^138^Ce systematics is hampered by their poorly constrained behavior during mantle melting, where La–Ce behave highly incompatibly and modeled La/Ce is extremely dependent on melt porosity. As shown in *SI Appendix*, Fig. S13, our modeling results are in good agreement with the SGR komatiites originating from 20 to 30% batch melting of a hybrid source that consists of depleted mantle and 10 to 20% lower-crustal restites. Moreover, our model can reproduce distinct Hf/Sm and Zr/Sm values prominent within SGR komatiites and their covariation with ^182^W ICs (Fig. 2 *A* and *B*). A recent study on mantle-derived rocks from the Kaapvaal Craton (17) reported a similar correlation of Hf/Sm with ^142^Nd compositions, arguing that this feature is unique to deep magma ocean crystallization processes that happened soon after Earth accretion. Our lower-crustal restite model can now offer an alternative explanation. The hybrid source model can also explain the positive initial γ^187^Os of the SGR komatiites [γ^187^Os = +3.7 ± 0.3 (30)]. As discussed in *Materials and Methods*, Re–Os systematics in our modeled reservoirs are more difficult to constrain, resulting in large propagated uncertainties for modeled ^187^Os compositions. However, our first-principle assumptions reveal that the addition of ca. 10 to 13% restite from Hadean protocrust to a depleted mantle source is in accord with previous models explaining positive initial γ^187^Os values in modern plume-related basalts and Archean komatiites by the presence of recycled eclogitic or pyroxenitic components in their mantle sources (49, 58). As for ^187^Os, modeling Pb isotopes, which are often used to assess crustal recycling in mantle sources, involves many uncertainties (e.g., hydrothermal redistribution of Pb) that irretrievably lead to large propagated errors in modeling approaches. Therefore, we conclude that Pb isotopes are not a diagnostic tool to identify Hadean crustal restites (see also *Materials and Methods*).

**Fig. 6.**
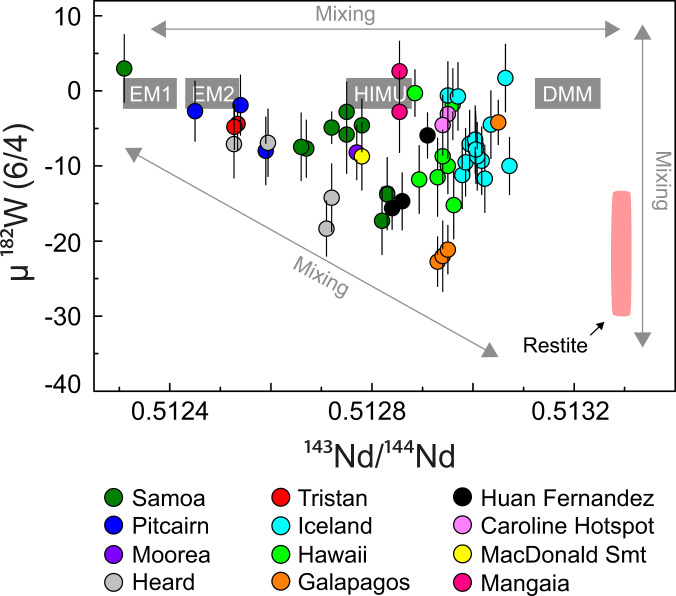
Compilation of combined ^182^W and ^143^Nd isotope data available for modern OIBs. Data were compiled from recent studies (8, 59) and references therein. Notably, the global compilation for modern OIBs shows a pattern similar to Archean mantle-derived rock assemblage from the Kaapvaal Craton, including an endmember with low µ^182^W and radiogenic ^143^Nd/^144^Nd. Also shown is the present ^182^W and ^143^Nd IC calculated for Hadean restites that remained isolated in the mantle (pink field). This global dataset can be best explained by the admixture of the classical mantle endmember components DMM, EM1, EM2, and HIMU to a primordial reservoir that is characterized by negative ^182^W anomalies and depleted ^143^Nd IC. A similar OIB compilation for ^182^W and ^176^Hf is limited by the availability of ^176^Hf isotope data but is shown for comparison in *SI Appendix*, Fig. S15.

In conclusion, the isotope patterns found here for Kaapvaal Craton rocks are clearly unique within the Archean rock record, and they may be locally restricted. Conceptually, however, the lower-crustal restite model for the Kaapvaal Craton presented in this study has global implications, as it provides an intriguing explanation of ^182^W isotope variations observed in modern OIBs. The model also provides additional constraints on the secular evolution of ^182^W isotope systematics in mantle-derived rocks through deep time. A recent study (59) has proposed that the global ^182^W dataset for OIBs can be explained by the admixture of the classical mantle endmember components DMM (depleted MORB mantle), EM1 (enriched mantle I), EM2 (enriched mantle II), and HIMU (high “μ” or ^238^U/^204^Pb) to a primordial reservoir that is characterized by negative ^182^W anomalies and depleted ^143^Nd/^144^Nd compositions. Remarkably, lower-crustal restites, formed between 4.35 and 4.25 Ga from ancient mafic protocrust, constitute a viable endmember for the global OIB array in μ^182^W–^143^Nd/^144^Nd space (Fig. 6), once calculated to present-day ^143^Nd/^144^Nd. We therefore speculate that lower-crustal restites from Hadean protocrust were delaminated and ultimately recycled into the mantle. Following recent models, such crustal remnants might have accumulated at the lower–upper mantle boundary and been incorporated into rising mantle plumes (50). Alternatively, they could have passed the lower–upper mantle boundary and descended into the lower mantle where they might have become part of large low shear-wave velocity provinces (LLSVPs), which are interpreted to contribute to rising mantle plumes (60). Indeed, it has been shown that restites modeled here have the potential to delaminate into the mantle, due to their density contrast compared to ambient mantle (52). Recent thermomechanical and thermodynamic modeling showed that garnet-rich assemblages will descend through the lower–upper mantle boundary and sink into the lower mantle (61). Accordingly, geophysical studies demonstrated that LLSVPs may represent mixtures of such recycled dense material that accumulated at the core–mantle boundary (62, 63). Taking into consideration that the strongly depleted restites would exhibit low He abundances, recycling of such components into the lower mantle would not significantly affect the ^3^He/^4^He ratios of a primordial undegassed host reservoir. Correspondingly, the observed coupled variations between ^3^He/^4^He ratios and µ^182^W (6, 8) would simply reflect variable proportions of such a hybrid lower-mantle reservoir in ascending mantle plumes. In this regard, our model provides an alternative explanation for the origin of negative ^182^W isotope anomalies in modern OIBs and bridges ^182^W isotope systematics in Archean mantle-derived rocks with observations from modern-day mantle plumes. Although ^182^W systematics in the mantle still need to be constrained in detail, the presence of small ^142^Nd and ^129^Xe isotope anomalies in OIBs (2, 7) clearly indicates the involvement of ancient mantle reservoirs that must have differentiated during the Hadean and remained isolated ever since. Our discovery of long-term preservation of Hadean protocrust in Earth’s mantle also has other far-reaching implications, in that its presence requires silicate reservoirs on Earth to have already differentiated during the lifetime of ^182^Hf (11).

## Materials and Methods

### Lower Hadean Protocrust Delamination Model.

The starting compositions, applied partition coefficients, respective mineral assemblages, references for decay constants and reservoir compositions, and calculations are listed in Dataset S3. In our model, we always used internally consistent sets of partition coefficients and assumed batch melting throughout (64). ICs for ^138^Ce, ^143^Nd, and ^176^Hf were modeled by using parent–daughter ratios from the calculated sources, using the appropriate decay constants (65–68), and assuming CHUR composition for the BSE (69, 70). The ICs for ^142^Nd and ^182^W were back-calculated by using the appropriate decay constants (71, 72), present-day IC for the BSE (73, 74), elemental Hf/W and Sm/Nd ratios for the BSE (25, 75), and solar system initials for the parent–daughter ratios (76, 77). Formation of a mafic protocrust is stage 1 of our model (Fig. 3*A*). The maximum age for the extraction of our protocrust is set by core formation, which could have been completed as early as 38 Ma after solar system formation (25). We assume extraction of mafic protocrust 50 Ma after solar system formation from a mantle with BSE composition (75). The timing of protocrust formation particularly affects the ICs of the short-lived isotope systems during further protocrust evolution (Fig. 5*A*). For protocrust formation, we used a consistent set of experimental partition coefficients for REE, HFSE, and Th assuming 20% batch melting at 2 GPa (41). Partition coefficients for W are rather incomplete in the literature. If not available, we calculated partition coefficients for W by using partition coefficients for mineral phases from experiments on garnet lherzolite (78) that were adjusted to the melt conditions in our model by using appropriate partition coefficients for Th. Both elements were shown to behave similarly incompatibly during silicate crystal–liquid fractionation (25). Correspondingly, the W/Th ratio of our modeled melts extracted from the primitive mantle (W/Th = 0.14) is indistinguishable from the canonical range reported in the literature (25).

At 4.35 Ga to 4.25 Ga (stage 2), we remelt our modeled mafic protocrust (Fig. 4*B*) and calculate [based on an experimental study (52)] the composition of a typical garnet-rich restite that remained after lower-crustal anatexis of a metamorphosed basaltic assemblage (estimated to be representative for the Hadean protocrust) at 12 kbar, in equilibrium with ca. 21% tonalitic melt. The timing of TTG formation as well as the residual mineral assemblage exerts a strong influence on the ^142^Nd evolution. In contrast, the ^182^W IC will not change, because the ^182^Hf–^182^W system went functionally extinct shortly after protocrust formation at ca. 60 Ma after solar system formation. Due to the enriched composition of the precursor and the long half-lives of their parent isotopes, prolonged tonalite formation will only cause small variations in isotopic ingrowth for ^143^Nd and ^176^Hf in the lower-crustal restites (Fig. 5*A*). Therefore, prolonged tonalite formation can explain decoupling of ^142^Nd from the other isotope systems in the residual garnet-rich restites and provides an explanation why ^143^Nd and ^176^Hf correlate so tightly with ^182^W but not ^142^Nd. Indeed, the occurrence of rare Hadean detrital zircons (56) and Hf isotope data in zircon reported for Paleoarchean gray gneisses of the eastern Kaapvaal Craton reveal incorporation of older continental crustal rocks with Eoarchean to Late Hadean age (53–55) that have not been directly preserved in the rock record of the Kaapvaal Craton. In addition, detrital PGM sampled from sedimentary units of the Kaapvaal Craton reveal redepletion ages of up to 4.1 Ga (79), hinting at remnants of Hadean protocrust within the Kapvaal Craton. Moreover, diamictites from the Kaapvaal Craton were found to preserve a negative ^182^W signature (57), hinting at upper continental crust with negative ^182^W composition. Assuming prolonged tonalite formation initiated by ca. 4.35 Ga and continued for 100 My, the variation of ^142^Nd IC within the tonalitic restites would be ca. 9 μ units at 3.55 Ga. In contrast, ^143^Nd and ^176^Hf would not vary by more than 1 ε unit.

Previous studies found evidence for incorporation of recycled mafic crustal material into melts derived from hybrid mantle plumes throughout Earth’s history (49, 58, 80, 81). Moreover, it has previously been shown that delamination of crustal restites into depleted mantle can cause melting of hybrid mantle sources and that the resulting trace element signatures resemble those of typical komatiites (47). Likewise, we propose here the mechanical incorporation of 10 to 20% of garnet-rich restites into an ascending plume that taps depleted mantle sources at ca. 3.55 Ga. Twenty to thirty percent of batch melting of such a hybrid source can reproduce the trace element compositions of the SGR komatiites (*SI Appendix*, Fig. S13). As previously suggested (12, 30), we attribute the variation within the SGR komatiite suite and their partially more depleted trace element compositions, compared to the modeled patterns, to olivine accumulation. For the upwelling mantle plume into which the restites were mixed, we assume 10% melt depletion at ca. 3.85 Ga. We use coherent, experimentally constrained melting parameters (41) to calculate the isotope and trace element composition at 3.55 Ga, the age of our samples. The modeled depleted mantle displays initial ε^143^Nd and ε^176^Hf values of +4.3 and +7.9, respectively, which is in perfect agreement with the modern terrestrial Hf–Nd array (36), within the range of the modeled DMM composition at 3.55 Ga (82) and also consistent with observational constraints from mantle-derived rocks from the Kaapvaal Craton (39). The ICs for ^182^W, ^142^Nd, ^143^Nd, and ^176^Hf of 20 to 30% melts extracted from a hybrid plume source, containing 10 to 20% of restite and corresponding proportions of plume-derived melt, is in accord with the range of ICs observed in SGR komatiites (Fig. 5*B* and Dataset S3). It is noteworthy that the ^182^W IC of the melt is controlled by the restite, because high modal abundances of garnet and amphibole, together with refractory Ti-rich phases (rutile/ilmenite), result in high bulk partition coefficients for W. This buffers the ^182^W IC against possible variations in the ambient Archean mantle [on average, ca. +13 ppm (14, 23)]. Variable proportions of rutile or ilmenite as a residual Ti-rich phase in the restites do not significantly affect the results of our model. Ratios of Nb/Ta have been proven to be valuable indicators to discriminate between rutile and ilmenite (83), but, unfortunately, no Ta concentrations are available for SGR komatiites. We expect ilmenite to be present in the restites, as this results in reasonable Nb/Th ratios (Nb/Th = 14.8 to 15.1) that are similar to the range observed in the SGR komatiites (Nb/Th = 11.2 to 14.6). Evidence for the presence of lower-crustal restites in the mantle source of the SGR komatiites is also provided by Zr/Sm (and Hf/Sm) ratios that are best explained by fractionation of garnet. The covariation of Zr/Sm (and Hf/Sm) with ^182^W IC is perfectly reproduced by our model (Fig. 2*B*).

The hybrid plume model can also explain the ^187^Re^187^Os isotope inventory of the SGR komatiites. The komatiite lavas from Schapenburg exhibit overall low PGE abundances and sample a melt-depleted, sulfur-exhausted mantle source (30). However, the positive initial γ^187^Os of the SGR komatiites (γ^187^Os = +3.7 ± 0.3) require that their mantle source evolved with a time-integrated suprachondritic Re/Os (30). A conceptual model for ^187^Os isotope systematics is presented in Dataset S3 applying the same time evolution path as for the other decay systems (Fig. 5). In brief, we modeled the ^187^Os IC of a melt that derived from a depleted mantle source and assimilated delaminated restite which remained behind after TTG melt extraction of a Hadean protocrust. The Re and Os abundances and ^187^Re/^187^Os of the Hadean protocrust are difficult to estimate because Re and Os can be highly variable within metamorphosed mafic crust, ranging from ca. 2 pg/g to 1,700 pg/g Re and 0.9 pg/g to 12 pg/g Os, yielding ^187^Re/^187^Os from ca. 5 to 2,700 (84). To circumvent this uncertainty, we used average Re–Os compositions of typical flood basalt samples from the Otong Java Plateau (Kwaimbaita Formation) derived from a primitive mantle source by magmatic differentiation, yielding ca. 1.2 ppb Re and 0.06 ppb Os and an average ^187^Re/^188^Os of 90 (80). Using these Re–Os abundances, the Hadean protocrust had developed a highly radiogenic γ^187^Os between ca. 2,800 and 4,400 until partial melting between 4.35 and 4.25 Ga (TTG melt formation). We assume that most of the Re in the protocrust was extracted during TTG formation through complete sulfide consumption (85). However, trace amounts of Re may be held back in residual garnet, where Re is compatible (86, 87). Considering previous melt depletion for the plume-related mantle reservoir, the Re budget of the modeled hybrid reservoir can be assumed to be fully controlled by the restite. We therefore use the average Re concentration in the SGR komatiites [36 ppt (12, 30)] as a minimum estimate for the Re concentration of the restite. Further, we assume that Os within the restite was fully retained by accessory chromite and magnetite (88). Correspondingly, after TTG formation, the restite retained a radiogenic γ^187^Os(3.55) between ca. 2,950 and 4,450, when applying a ^187^Re/^188^Os of 2.7.

Our constraints from Ru isotopes show that the PGE inventory in the SGR komatiites does not reflect a mantle source that lacks significant amounts of late-accreted components. More likely, the overall low PGE abundances reflect residual PGM in a melt-depleted source that lead to very low ^187^Re/^187^Os ratios (≤0.005) assuming low Re concentrations (ca. 0.001 ng/g) and depleted mantle–like Os compositions [0.8 ng/g to 9 ng/g (89)]. Considering that melt depletion occurred at 3.85 Ga, this mantle source would develop to a γ^187^Os of ca. −21 at 3.55 Ga. As previously proposed, the SGR komatiite lavas contain ca. 1.1 ng/g Os (30). This comparably low concentration can be explained if PGM or refractory alloys remained in the sulfur-exhausted source (30) holding back a large amount of Os–Ir–Ru. Our model calculations show that the assimilation of ca. 10 to 13% restite to the komatiite melt can reproduce the radiogenic γ^187^Os values observed in the SGR komatiite suite (Dataset S3 and *SI Appendix*, Fig. S9).

Similar to the other decay systems, the involvement of a TTG formation event during the Hadean is necessary to explain the γ^187^Os values of the Schapenburg komatiites at 3.55 Ga (Dataset S3). Without such an event, the protocrust would have developed to extreme γ^187^Os values of ca. 15,000, which would dominate the SGR komatiites. These conceptual assumptions are also in accord with other plume-derived magmatic systems where radiogenic Os ICs have been interpreted as being derived from hybrid plumes that incorporated a pyroxenite or eclogite component (49, 58, 80).

As an additional constraint to assess incorporation of Hadean restites in the komatiite melt, we also modeled the Pb IC. However, it is difficult to evaluate whether these Hadean restites that were recycled into the mantle carry a diagnostic Pb IC. From mineral partition coefficients alone, it is expected that restites complementary to TTGs would exhibit strongly anomalous Pb ICs. By a “first-order” estimate (for calculations, see Dataset S3), we assume a single-stage Pb evolution starting at 4.567 Ga with a μ (^238^U/^204^Pb) of 8.5 and an α_0_ (^206^Pb/^204^Pb_initial_) of 9.307 (90). Restites that remained after partial melting of a mafic protocrust (TTG formation at 4.35 Ga) would develop toward an unradiogenic ^206^Pb/^204^Pb composition due to a low μ^238^U/^204^Pb of ∼3.7 and display a present-day ^206^Pb/^204^Pb of only ∼14.3. The complementary TTGs would exhibit an elevated μ^238^U/^204^Pb of ∼10.2 and evolve toward radiogenic present-day ^206^Pb/^204^Pb compositions (∼22.1). However, Pb isotope systematics during partial melting of hydrated oceanic crust are not only controlled by mineral partition coefficients, as the elements involved (U, Th and Pb) display different redox sensitivities and reveal a different mobility in the presence of fluids (91). It is therefore very likely that U–Pb isotope systematics were often affected by ocean floor processes, resulting in highly variable initial Pb ICs in Archean TTGs and their mafic counterparts. Indeed, while many Archean cratons have preserved a long-lived high-μ continental lithosphere with distinctive Pb ICs (92), other cratons show large Pb isotope variations with more unradiogenic Pb isotope patterns (93–95).

### Calculation of BGGT Mantle Source Averages.

Postemplacement processes such as hydrothermal alteration or metamorphism may have potentially overprinted the isotope and trace element composition of individual samples and obscured the primordial information on their parental sources. For long-lived radiogenic nuclides, the closest approximation to the mantle source composition is to calculate a mean of the initial ratios for individual samples. The initial ratios were determined using accepted emplacement ages and the measured ICs of daughter (e.g., ^176^Hf/^177^Hf) and parent isotopes (e.g., ^176^Lu/^177^Hf) for each individual sample. In the case of large sample numbers, outliers with disturbed ICs can be identified using isochron plots. By this approach, we determined the initial ɛ^143^Nd and ɛ^176^Hf IC for the mantle source of magmatic rocks from the Komati Formation. Using average initial ɛ^143^Nd and ɛ^176^Hf values (18) and using the accepted emplacement age of 3.482 Ga (96), we calculated ^147^Sm–^143^Nd and ^176^Lu–^176^Hf reference isochrons for ultramafic and mafic rocks from the Komati Formation mantle source. Komati Formation samples from this study that do not plot on reference isochrons for ^147^Sm–^143^Nd (*SI Appendix*, Fig. S10*A*) and ^176^Lu–^176^Hf (*SI Appendix*, Fig. S10*B*) were regarded as being significantly disturbed (CGN BARB 1.9, 1.14, and 2.6 for ^143^Nd, and CGN BARB 1.3, 1.14, 2.2, and 2.6 for ^176^Hf). Excluding these samples results in regression ages of 3,566 ± 76 Ma for ^147^Sm–^143^Nd (*SI Appendix*, Fig. S10*A*, MSWD 1.3) and 3,457 ± 300 Ma (*SI Appendix*, Fig. S10*B*, MSWD 4), respectively, overlapping with the excepted emplacement age (3,482 ± 5 Ma) obtained from single zircon U–Pb ages (96). Average initial ICs of these least-altered samples provide the best estimate for the IC of their common mantle source at 3.482 Ga and yield robust initial ɛ^143^Nd of +0.7 ± 0.1 (95% CI) and ɛ^176^Hf of +3.0 ± 0.3 (95% CI). In the case of other units from the Kaapvaal Craton investigated in this study (Sandspruit, Theespruit, and Dwalile Formations), datasets are limited to two to three cogenetic samples. Although these small sample numbers preclude an isochron approach, we think that their mantle source is still best estimated by averaging data of these two or three samples. In these cases, uncertainties are given by the corresponding 2 SD. For the same reasoning, the best estimate for the ^182^W IC of the Komati, Sandspruit, Theespruit, and Dwalile mantle sources is given by the average of data provided in this study, with corresponding 2 SD as uncertainties. For the Schapenburg komatiite suite, we used source compositions provided in the literature (12). Source ratios of Hf/Sm and Zr/Sm are estimated based on average values for samples investigated in this study (Komati, Dwalile, Sandspruit, and Theespruit) and previous publications [Schapenburg (12)]. A compilation of best estimates for mantle source compositions from the Kaapvaal Craton is provided in Dataset S2.

### Assessment of Magma Ocean Models Involving Perovskite Fractionation.

Alternative models have been proposed for the origin of the SGR komatiites (12), involving fractionation of a high-pressure and high-temperature Mg- and Ca-perovskite mineral assemblage in an early terrestrial magma ocean. In short, we found that such models are highly dependent on the sets of partition coefficients used and the choice of Ca:Mg-perovskite assemblages. Independent of this issue, some important diagnostic features of the samples analyzed here (e.g., ^142^Nd–^176^Hf–^143^Nd isotope relationships) cannot be reproduced by a magma ocean model or do not require the presence of perovskite cumulates at all.

In detail, we modeled evolution of a mantle reservoir that has undergone perovskite segregation, tightly following a previous model for the SGR (12). Herein, a primitive mantle undergoes removal of 10% of the perovskite (5:95% Ca:Mg-perovskite) at 4.537 Ga before it evolves until 4.027 Ga. Subsequently, this reservoir undergoes batch melting in the spinel stability field at 4.027 Ga before it melts at 3.55 Ga to produce the SGR komatiites. For perovskite, we used a more rigorous, internally consistent set of partition coefficients from ref. 40, using laser ICPMS data for their representative experiment H2020 a+b, and relative abundances of Mg- and Ca-perovskite of ref. 1). Mantle depletion at 4.027 Ga was modeled in analogy to the parameters presented by ref. 12, but again using more updated sets of partition coefficients (41). All other parameters, like decay constants or CHUR values, are as above. We refrained from modeling W because the original dataset for perovskite (40) does not include W partition coefficients. Previous modeling (12) referred to lattice strain modeling of D_W_, but the lattice strain model used by ref. 40 is only applicable to 1+, 2+, 3+, and 4+ ions. Recent work (97, 98) has shown that the valence state of W, even in the more reduced regime of an early magma ocean, is rather 6+.

Hafnium–Nd–Ce modeling results are shown as blue symbols in Fig. 3, recalculated to 3.55 Ga. It is important to note that the fractionation of perovskite during magma ocean crystallization does not lead to suprachondritic Lu/Hf and to a decoupling of ε^143^Nd and ε^176^Hf systematics, once recalculated to 3.55 Ga. Rather, the Hf–Nd composition of the modeled mantle after perovskite segregation is near-chondritic at 3.55 Ga. Depletion of such a mantle reservoir at 4.027 Ga in the spinel stability field yields decoupled Hf–Nd ICs, but at extremely radiogenic ε^143^Nd at a given ε^176^Hf (Dataset S3), which is nowhere found in our sample set. Larger amounts of residual garnet during mantle depletion at 4.027 Ga may result in ε^143^Nd–ε^176^Hf systematics that resemble the compositions of SGR komatiites, in analogy to the modern-day terrestrial mantle array (36). Rather, this finding is in line with the consideration that the origin of the terrestrial Hf–Nd mantle array has already been established in early Earth as a consequence of deeper-mantle melting and an increased role of residual garnet or recycling of garnet-bearing restites (37). Moreover, the choice of Ca–Mg-perovskite assemblages exerts a strong influence on the decoupling of ^143^Nd and ^176^Hf isotope systematics (24). To better illustrate these effects, we show the evolution of modeled SGR komatiite source compositions in ^143^Nd–^176^Hf and ^143^Nd–^138^Ce space in response to variable Ca–Mg-perovskite assemblages (Ca:Mg-perovskite from 20:80 to 0:100). It becomes apparent that the decoupling of ^176^Hf from ^143^Nd strongly depends on the amount of Ca-perovskite crystallizing together with Mg-perovskite. However, the coprecipitation of Ca- and Mg-perovskite during an initial 10% of fractional crystallization remains an open issue, as Ca-perovskite does not appear as first liquidus phase at lower-mantle conditions (43, 44). Most importantly, the model cannot explain why ^142^Nd compositions in mantle-derived rocks from the Kaapvaal Craton do not correlate with ^143^Nd–^176^Hf compositions.

A popular way to verify whether perovskite fractionation took place is to inspect trace element ratios that behave sensitively to perovskite fractionation. However, as Ca-perovskite fractionates many trace elements in the opposite way from Mg-perovskite (40), many of the geochemical signatures often referred to are actually nondiagnostic. For example, Hf/Sm in rocks from the Kaapvaal Craton were taken as evidence supporting the hypothesis that ^142^Nd anomalies result from fractionating perovskite in a deep magma ocean (17). However, when considering different proportions of Ca–Mg-perovskite and taking into consideration that the absolute amount of fractionated perovskite may vary, it is possible to generate a large range of Hf/Sm ratios (*SI Appendix*, Fig. S11*A*). This clearly demonstrates that trace element ratios should be used that are largely insensitive to the choice of Ca–Mg-perovskite proportions (e.g., Zr/Nb). However, models that only fractionate a small fraction of perovskite (12) do barely fractionate such element ratios, thus withstanding such investigations (*SI Appendix*, Fig. S11*B*).

Collectively, our modeling of a mantle reservoir involving perovskite segregation and subsequent mantle depletion demonstrates that ^143^Nd–^176^Hf isotope systematics are nondiagnostic features to identify perovskite fractionation and cannot explain the full range of ICs found in our sample set and previously published isotope data for the SGR komatiite suite.

### Analytical Protocol.

Our analytical protocol for ID analysis follows procedures that were described in detail by previous studies (23, 99, 100). For ^138^La–^138^Ce measurements, we processed 1 g of sample powder. For La–Ce ID measurements, a 5% aliquot was spiked with a ^138^La–^142^Ce isotope tracer. For the 95% aliquot, we utilized the first-stage cation resin column of a previously published protocol for W(14) to separate REE from matrix elements for high-precision ^138^Ce IC measurements. This step is required since sample loads larger than 200 mg exceed the capacity of the first-stage column in our ^138^La-^138^Ce separation protocol (100). Measurement protocols for La–Ce ID measurements as well as for Ce IC measurements followed a previously described routine (100) except that 10^12^ Ω resistors used for interference corrections were replaced by 10^13^ Ω resistors. All data were normalized relative to ^136^Ce/^140^Ce of 0.002124072 (101) and are given relative to a ^138^Ce/^136^Ce value of 1.33738 for the Mainz AMES standard solution (102). All samples were analyzed repeatedly. Reported uncertainties either refer to the corresponding 95% CI (*n* ≥ 4) or to our intermediate precision (±0.21 ɛ-units) (20).

Details about the chemical separation and purification protocol of Ru are described elsewhere (22) and involved NiS fire assay digestion, cation column chemistry, and microdistillation. High-precision Ru IC measurements were conducted on a Thermo Fisher Neptune Plus MC-ICP-MS at University of Cologne following a previous protocol (22). In short, ∼100 ng/mL solutions were introduced at an uptake rate of ca. 50 μL/min using a PFA nebulizer and a Cetac Aridus II desolvating system. Measurements comprised 100 integrations of 8.4 s and were preceded by an on-peak baseline (40 integrations of 4.2 s) on a solution blank (0.28 M HNO_3_). The data were internally corrected for mass bias by using ^99^Ru/^101^Ru = 0.7450754 and utilizing the exponential law. Sample solutions were always bracketed by measurements of a concentration-matched Ru standard solution (Alfa Aesar Ru) to report relative Ru ICs in the μ-notation, which gives the parts per million deviation for ^i^Ru/^101^Ru isotope ratios between a sample and bracketing standard solutions. The accuracy of the Ru isotope measurements was evaluated by the repeated analysis of replicate digestions of a 2.05-Ga chromitite from Bushveld igneous complex (UG-2) and two 3.8-Ga chromitites from the Itsaq gneiss complex, SW Greenland (samples 194856 and 194857), that were previously shown to display modern mantle–like and anomalous Ru ICs, respectively (22). Our Ru isotope data obtained for all three chromitites agree well with previously reported data (22) (*SI Appendix*, Fig. S6). The uncertainty for measurements is either given as the external uncertainty of the method (22) (2 SD for samples measured *n* < 4 times) or the corresponding 95% CI (if *n* ≥ 4).

High-precision ^182^W isotope measurements mainly followed established analytical protocols (14, 23) that were slightly modified to yield highly purified W solutions from large sample loads (up to 18 g) and to improve our analytical uncertainty. In short, samples were measured at average signal intensities of 17 V for ^182^W (using 10^11^ Ohm amplifiers) corresponding to a ∼175 ng/mL W sample solution at an uptake rate of ca. 55 μL/min. Samples were always bracketed by a concentration-matched certified reference material (National Institute of Standards and Technology [NIST] SRM 3163). Results of high-precision W isotope analyses are reported in the μ-notation (equivalent to parts per million) relative to the bracketing NIST solutions and always refer to the measured ^182^W/^184^W ratio that has been corrected for mass bias by using ^186^W/^184^W = 0.92767 (103). All samples were repeatedly analyzed (*n* = 6 to 11), and uncertainties for average W ICs are correspondingly reported as 95% CI (Dataset S1).

Our protocol for the chemical purification of W for high-precision IC analysis comprises four columns. During a cation (AG 50 W-X8 resin, column I) and anion exchange stage (AG 1-X8 resin, column II), W is separated from matrix elements and HFSE and Ti, respectively. Columns III (TEVA resin) and IV (TODGA resin) are clean-up columns that yield purified W cuts. In this regard, the repetition of the final-stage column during the chemical separation of W (23) improves the purification from remaining matrix elements. The final W-bearing eluate was directly loaded onto BioRad Poly-Prep columns filled with 0.8 mL of Eichrom prefilter material to extract organic compounds. This, together with threefold treatments with 80 µL of cHNO_3_–30%H_2_O_2_ at maximum 60 °C after dry-down steps during and after the chemical separation, strongly improved yields and removed mass-independent effects on ^183^W (14). Prior to loading onto our final-stage column, we combined up to 10 cuts, in case sample powders were split up into aliquots (up to 1.3 g) during matrix separation. The combination of sample solutions during chemical separation does not affect the accuracy of our high-precision ^182^W isotope analysis, as demonstrated by indistinguishable results for sample solutions of our in-house rock reference material LP 1 (historical La Palma Basalt), that were either obtained from single column cuts (up to 1.3 g) or combined solutions from 10 column cuts (in total, 11.3 g). The purpose of combining the final cuts is to efficiently measure the cuts by reducing the cumulative volume of leftovers after multiple measurements of individual solutions. This allows measuring at the highest beam intensities possible and, together with our refined separation procedure, significantly improves the analytical uncertainty of our measurements. This is also reflected by the intermediate precision of our in-house rock reference materials LP 1 and AGC 351 that were always measured in every session, yielding markedly improved 2 SD of ±1.5 ppm and ±2.7 ppm, respectively (*SI Appendix*, Fig. S1). The µ^182^W session averages for LP 1 (1480 OIB from La Palma) and AGC 351 (3,455-Ma gneiss from Swaziland) overlap within their 95% CI (LP1 = −0.4 ± 1.0 ppm and AGC 351 = −0.2 ± 0.5 ppm) and are indistinguishable from the NIST reference material and previously reported long-term averages for the same sample powders (14, 23). Additionally, we also performed repeated analyses (*n* = 15) of a 3.27-Ga-old komatiite (sample 160245, Ruth Well Formation) from the Pilbara Craton Western Australia that exhibits highly elevated W concentrations of 19.1 μg/g (23). The µ^182^W session average for sample 160245 (µ^182^W = +7.9 ± 0.7 ppm, 95% CI) is in agreement with previous results (23) and shows a good intermediate precision (2 SD of ±2.5 ppm). This, together with the elevated ^182^W IC and high W concentration of sample 160245, validates the method for analytical campaigns addressing ^182^W isotope systematics in Archean mantle-derived rocks that often display anomalous ^182^W ICs.

## Supplementary Material

Supplementary File

Supplementary File

Supplementary File

Supplementary File

## Data Availability

All study data are directly included in the article and/or supporting information.
