# Supplementary material for: Long-term preservation of Hadean protocrust in Earth’s mantle
Source: Proc Natl Acad Sci U S A. 2022 Apr 22;119(18):e2120241119. doi: 10.1073/pnas.2120241119 (PMC9170142; doi:10.1073/pnas.2120241119)
Supplement: Supplementary File [file pnas.2120241119.sapp.pdf]

SI Appendix

Geological background of our sample selection

We analyzed a comprehensive set of rocks from the Kaapvaal Craton that range from different types of grey orthogneisses (TTGs and more evolved granitoids) to mantle-derived lithologies of mafic-ultramafic composition. This representative suite of 17 samples span an age range from 3.55 to 3.22 Ga and represent the main lithological units of the Ancient Gneiss Complex (AGC), also comprising the oldest mafic rocks (lower Onverwacht Group, 3.55 to 3.45 Ga) of the Barberton Granite-Greenstone Terrane (BGGT).

The AGC is located in Swaziland and is a typical high-grade gneiss terrain that comprises 3.66-3.20 Ga old rocks(1). The oldest part of the AGC comprise polydeformed granitoid gneisses, heterogeneous in age and composition(2, 3), that are interbanded with amphibolites. Together, they formed layered grey gneiss sequences in response to ductile deformation under high strain conditions(1). The different varieties of rocks from this sequence have been summarized as the Ngwane Gneiss (NG)(4). The oldest generation of NG (NG *sensu stricto)* are 3.66 Ga to 3.5 Ga granitoid gneisses(2, 3, 5–7) that mainly belong to the tonalite-trondhjemite-granodiorite (TTG) suite but also comprise granitic rocks. As indicated by trace element systematics(5), whole rock Nd isotope systematics(8) and Hf-in-zircon isotope data(2, 5, 6) the protoliths of the orthogneisses resulted, at least in part, from melting of a LREE enriched source with considerable residence time, most likely older continental crust of Eoarchean to late Hadean age. Younger generations of grey gneisses, which are mapped as NG, were emplaced after 3.45 Ga. These show the same field appearance as the 3.66-3.45 Ga NG but are as young as 3.2 Ga(8, 9). The oldest NG hosts scattered remnants of supracrustal assemblages with greenstone belts (e.g. Dwalile Supracrustal Suite, DSS(10)). These remnants postdate the oldest NG, vary in size and are either infolded, occur as tectonically intercalated xenoliths of a few centimeters or even represent coherent blocks of several kilometers(10, 11). The origin of these remnants remains contentious. They were interpreted either as strongly flattened dikes(10, 12) or as dismembered portions of the Dwalile Greenstone Remnant (DGR), which represents the largest of the greenstone remnants of the AGC(1, 2, 11, 13). The DGR is located in SW Swaziland and the supracrustal rock assemblage (metavolcanics, metasediments) were shown to be extruded between 3.44 and 3.46 Ga, therefore postdating the oldest generation of NG(11, 14, 15). Notably, the metavolcanic rocks from the DGR share geochemical similarities with volcanic assemblages from the Onverwacht Group which hints at a genetic link between the DGR and the BGGT(11, 12, 14). Based on trace element systematics and variable whole-rock initial εNd and εHf values it has been argued that the mafic and ultramafic DGR rocks were derived from a mildly depleted mantle source and were in part contaminated by rocks from an ancient continental source, presumably crustal material of NG-like composition(11, 14). The oldest NG and intercalated members of the DSS were intruded by the texturally and compositionally distinct Tsawela Gneiss between 3.48-3.43 Ga(6, 9, 10, 16, 17) and younger generations of grey gneisses that date back to ca. 3.2 Ga(9).

All sample localities are shown in SI Appendix, Fig. S13 and GPS coordinates are provided in previous studies(2, 13, 16, 18). We have analyzed two grey gneisses from

the >3.45 Ga NG suite that were collected along the Mtimane River in the Mankayane area in central Swaziland, where granitoid gneisses of different ages were variably affected by intensive regional migmatization at ca. 3.2 Ga(11, 19, 20). Both samples (AGC 351 and AGC 352) were previously described(2, 16). AGC 351 is a 3.455 Ga old, strongly migmatized grey gneiss of near granitic composition and interpreted to be derived from felsic crustal precursors that mixed with juvenile, depleted mantle-derived melts(2, 19). AGC 352 is a 3.442 Ga very homogeneous fine grained grey gneiss(16).

We have analyzed several samples from greenstone remnants that are interlayered with grey gneisses of the AGC. We investigated two komatiites and one amphibolite from the DGR (AGC 83, AGC 86 and AGC 38), one typical amphibolite fragment as found in the AGC (AGC 222) and a 3.455 Ga gabbroic enclave (AGC 350) from central Swaziland. The mafic-ultramafic rock samples from the DGR were previously characterized(11, 14). Sample AGC 222 is a fragmented amphibolite enclave from Kubuta in central Swaziland with a minimum age of 3.4 Ga(21). It is similar in composition to other greenstone remnants found in the AGC(2, 16). Gabbroic enclaves like AGC 350 can be found along the Mtimane River in the Mankayane area close to the sample localities of AGC 351 and AGC 352. As described by reference (19) the precursors of the gabbroic enclaves were emplaced together with granitoid gneisses at 3.455 Ga. At about 3.2 Ga, a tectono-magmatic-metamorphic event reworked the grey gneisses and greenstones(20) which led to boudinage and local anatexis of the gabbros and migmatization of the grey gneisses (e.g. sample locality of AGC 351).

The youngest samples from the AGC are two ca. 3.2 Ga gneisses. Sample AGC 473 is a 3.24 Ga grey gneiss of trondhjemitic composition, which intruded into the oldest generation of NG northwest of the DGR. Based on structural considerations, the adjacent NG were interpreted as basement for the volcanic sequences of the DGR(10). Our younger grey gneiss sample AGC 473 belongs to the youngest generation of NG but contains inherited zircon grains of 3.49 Ga and ca. 3.64 Ga(22). This young generation of grey gneisses belongs to a 3.2 Ga magmatic event that is typically associated with indicators for strong deformation and high-grade metamorphism and therefore suggested to be the result of migmatization and crustal melting of older generations of crustal rocks(13, 19). Sample AGC 445 is a 3.216 Ga old grey gneiss from the Piggs Peak area also belonging to the former 3.2 Ga NG generation(13).

The AGC is in faulted contact with the BGGT along the ca. > 3.2 Ga old Phophonyane shear zone northwest of Pigg´s Peak town(7) and is spatially separated by sheet-like intrusions of the Mpuluzi and Piggs Peak batoliths. Rocks from the BGGT comprise a complex association of greenstone sequences and grey gneisses. The greenstone sequences in the BGGT (referred to as the Barberton Greenstone Belt, BGB, or the Barberton Supergroup) comprise a complex association of volcanic-sedimentary rocks that were deposited over more than 300 million years from < 3547 to > 3219 Ga(23). The volcano-sedimentary sequence of the BGB has traditionally been divided (from base to top) into three main lithostratigraphic units: The Onverwacht, Fig Tree, and Moodies groups. The Onverwacht Group (OG) is the oldest succession of the BGB and comprises voluminous mafic to ultramafic metavolcanics successions with sparsely interbedded metasediments. As we only analyzed samples from the lower OG, we provide only a short overview about the lowest stratigraphy of the BGB. The OG is subdivided into the lower and upper Onverwacht Group, marked by a chert layer,

known as the Middle Marker. The lower OG comprises the Sandspruit, Theespruit, and Komati Formations, the upper OG includes the Hoggenoeg, Noisy, Mendon, and Kromberg Formations(24). The oldest magmatic events preserved in the lithostratigraphic succession of the BGB are mafic-ultramafic and felsic metavolcanic rocks. This bimodal sequence (originally assigned to the Sandspruit and Theespruit Formations) comprises the oldest rocks of the lower Onverwacht Group. The metavolcanic rocks of the Sandspruit and Theespruit Formations were shown to be time-equivalent and deposited during one single volcanic event at ca. 3530 Ma and therefore constitute a single lithostratigraphic unit(25). The record of the somewhat younger 3.482 Ga Komati Formation(24) bears witness to a period of prolonged volcanic activity, as it comprises a continuous succession of alternating komatiitic, komatiitic basalt, and tholeiitic basalt lava flows without any intercalated sedimentary layers that would reflect a hiatus in the stratigraphy(26).

The BGB is surrounded by 3.521 to 3.197 Ga old granitoid gneisses(13) that form a cluster of 12 diapiric plutons with a wide variety of compositional types that intruded into the lowermost formations of the BGB(27). They can be subdivided into two major compositionally families that were emplaced during two periods: The older (3.45-3.2 Ga) TTG group that was coeval with deposition of supracrustal sequences in the BGB, and the much younger (ca. 3.1 Ga) GMS group (granite-monzonite-syenite) which intruded after sedimentation and stabilization of the crust through continued deformation of the TTG basement and greenstone sequences at ca. 3.2 Ga(23).

Our samples were collected at the southwestern margin of the BGGT southeast of the town of Badplaas, in an area around the settlement of Tjakastad (SI Appendix, Fig. S13). Here a significant proportion of the metavolcanic rocks from the Sandspruit and Theespruit Formations occur as dismembered rafts and xenoliths in tonalitic-trondhjemitic gneisses of the Badplaas, Stolzburg and Theespruit Plutons in the southern part of the Barberton Mountain Land(27, 28).

In order to better understand the depletion history of the Kaapvaal Craton we also investigated ultramafic rocks from the Komati Formation sampled from the BARB1 and BARB2 drillcores that were drilled during an International Continental Drilling Program (ICDP-2009/01, Exp.ID 5047) in the Onverwacht Group of the BGB(29). The exact core-positions of the samples analyzed are provided in Dataset S2.

Supplementary Figures

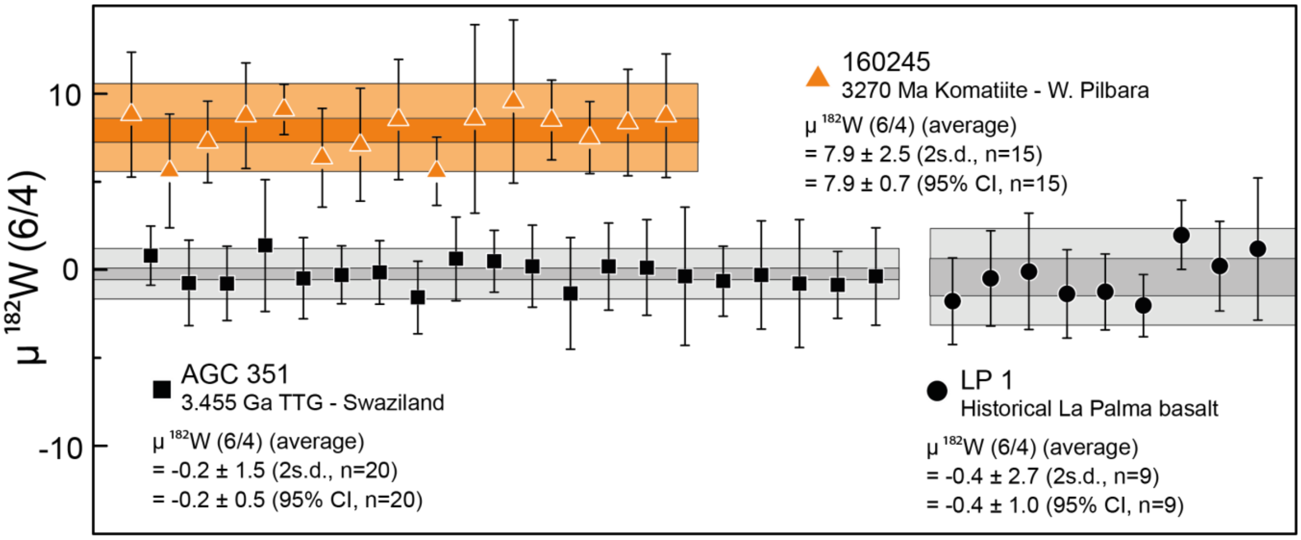

Fig. S1: Intermediate precision for $\mu^{182}W$ (6/4), inferred from the repeated analysis of multiple digestions for our in – house reference materials AGC 351, LP 1, and 160245 that are reported relative to W NIST SRM 3136. Each symbol refers to the average value of multiple measurements conducted during an analytical session. The uncertainties for the session mean values are given by the corresponding 95% CI. The intermediate precision for our in–house reference materials are given by the 2 SD of the session mean values.

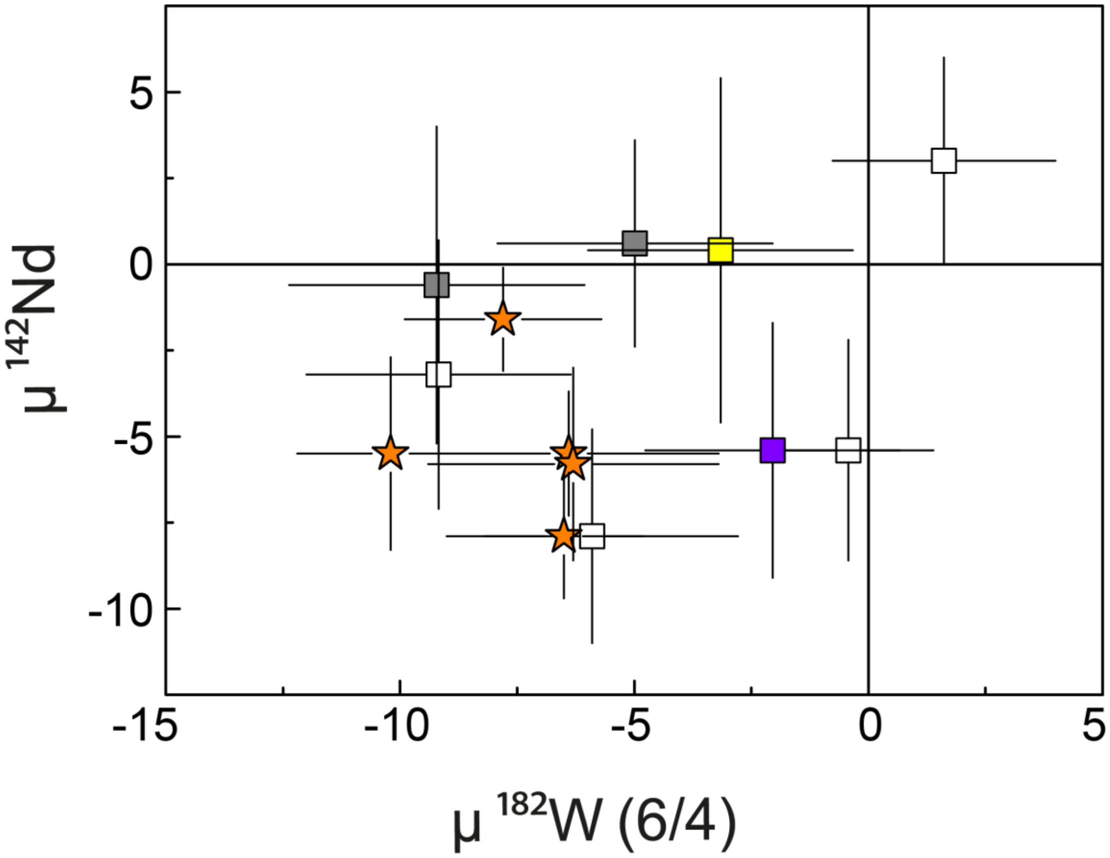

Fig. S2: Compilation of available μ^{182}W and μ^{142}Nd for crustal and mantle-derived rocks from the Kaapvaal Craton. Symbols for mantle-derived samples are the same as in Fig. 1. Crustal rocks are shown as open symbols. The ^{142}Nd isotope compositions for samples from our study were presiously reported(22) and combined ^{182}W – ^{142}Nd systematics for komatiites from the Schapenburg Greenstone Remnant (SGR) were taken from the literature(30). The combined data show a tendency towards negative anomalies but reveal no clear correlation due to the comparatively large uncertainties.

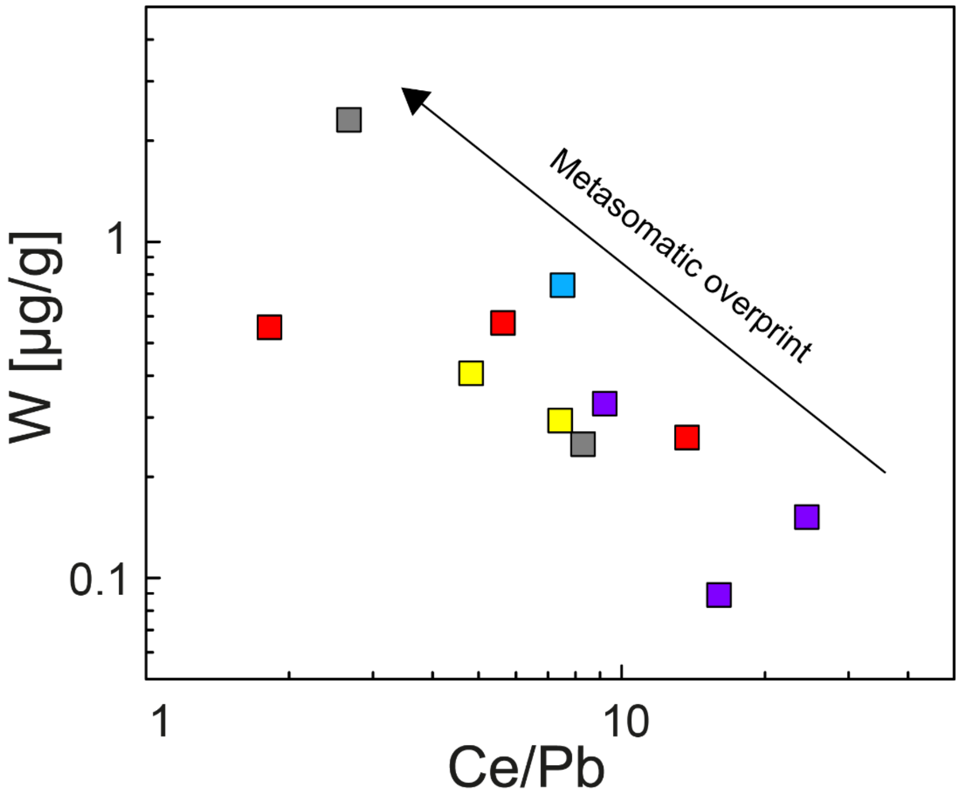

Fig. S3: Compositions of W (µg/g) vs. Ce/Pb as an indicator for selective W mobility by metasomatic fluids. Symbols are the same as in Fig. 1.

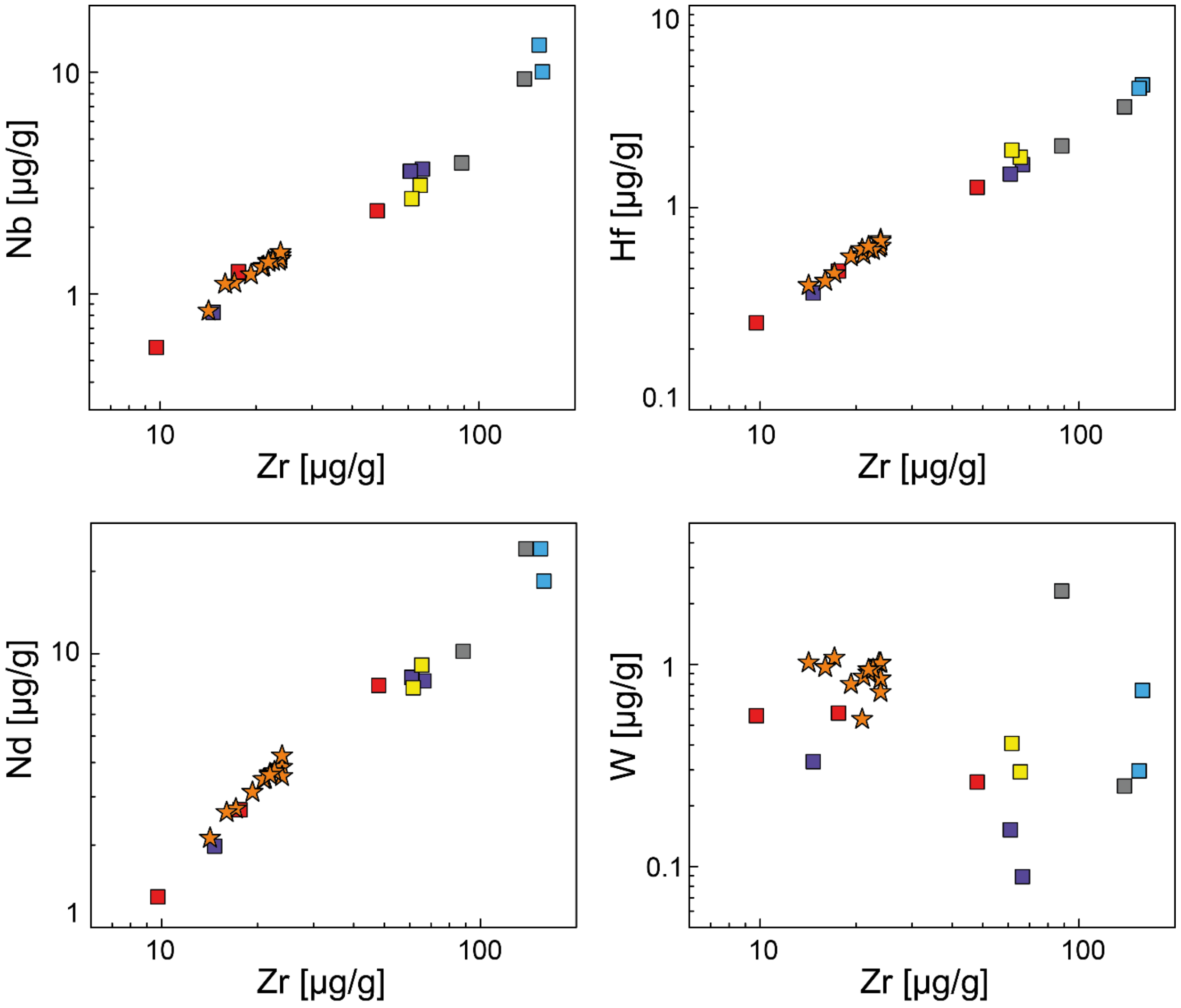

Fig. S4: Trace element variation diagrams [µg/g] of Nb, Hf, Nd, and W vs. Zr-content for our samples and the Schapenburg komatiite suite(30)**.** The positive correlations of incompatible elements (e.g. Nb, Nd, Hf) in variation diagrams vs. Zr content reveal that HFSE and REE were not affected by metasomatic processes. In contrast, no primary magmatic differentiation trends are preserved for W. Symbols are the same as in Fig. 1.

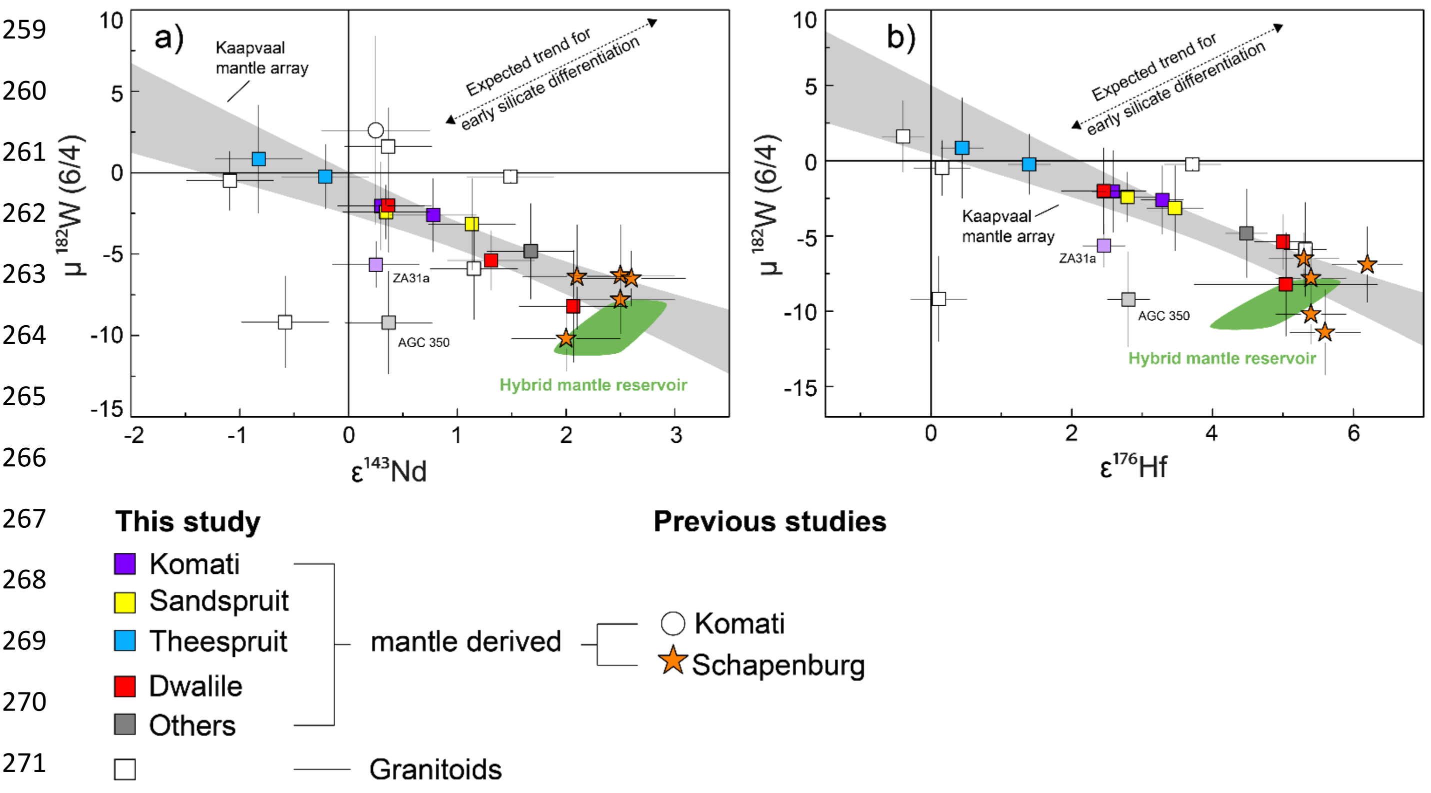

Fig. S5: Measured $\mu^{182}W$ vs. $\varepsilon^{143}Nd_{(t)}$ (a) and $\mu^{182}W$ vs. $\varepsilon^{176}Hf_{(t)}$ (b) for mantle-derived and TTG-like mafic rock samples from the Kaapvaal Craton including literature data. The literature data include previously published data for komatiites from the Schapenburg Greenstone Remnant (orange asterisks)(30) and the Komati Formation (open square)(31,32). We note that previously published literature data for the Komati Formation only report combined $\mu^{182}W$ vs. $\varepsilon^{143}Nd_{(t)}$ data for one single sample (sample BV 02, open square)(31,32). The green fields illustrate modeled values of our proposed hybrid reservoir (10-20% restites admixed to depleted mantle). The shaded grey field, referred to as Kaapvaal mantle array, is an uncertainty envelope employing the 95% confidence interval in which of all mantle-derived samples are expected to fall. Note, that the negative co-variation displayed by the Kaapvaal mantle array does not follow the expected trend for early silicate differentation (indicated by dashed lines).

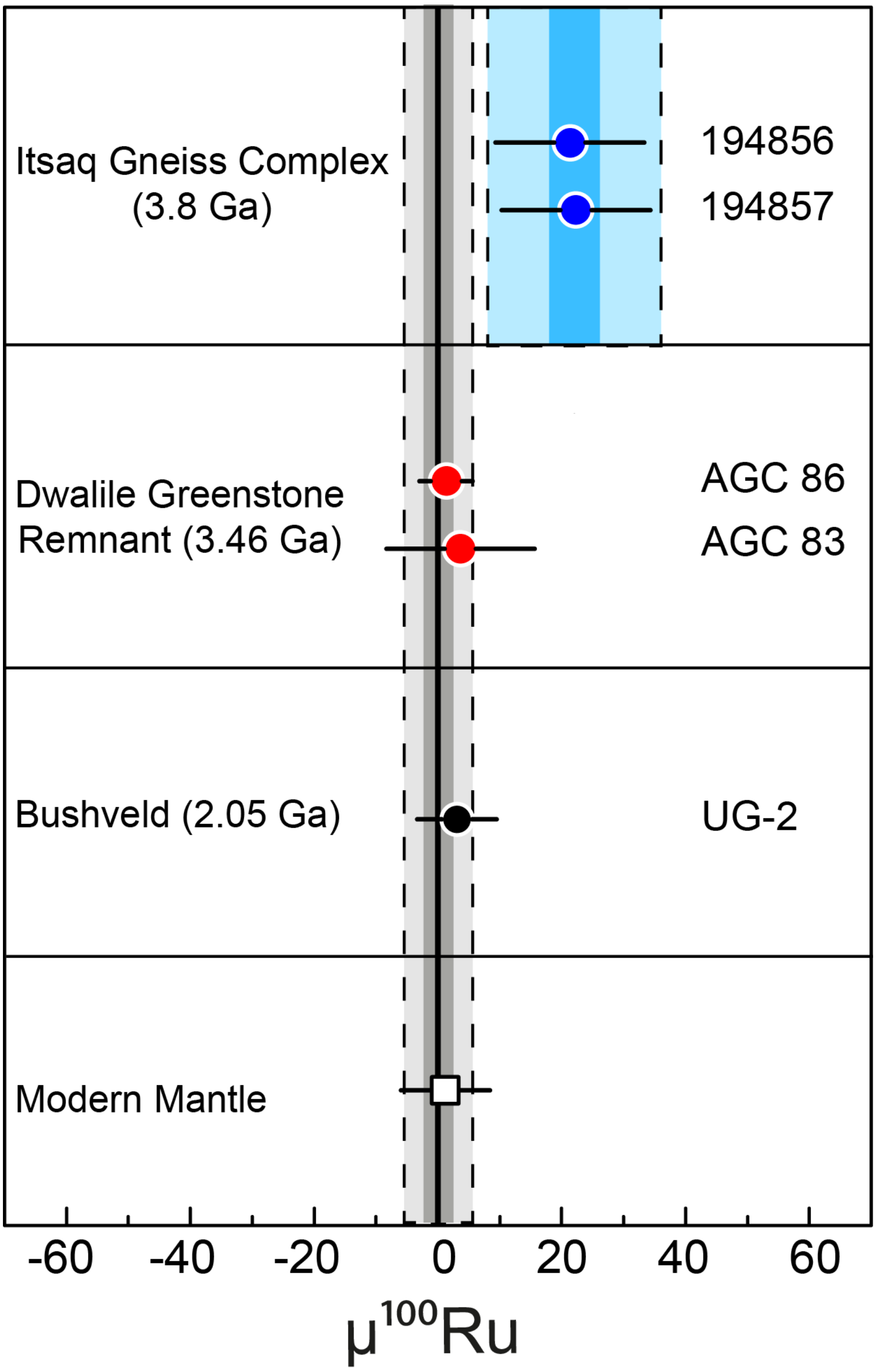

Fig. S6: μ^{100}Ru data for Archean and Palaeoproterozoic rocks measured in this study and a previous publication(33) compared to the modern mantle(34). Uncertainties for individual data points either refer to the external uncertainty of the method (2.s.d. for samples measured n<4 times) or involve the corresponding 95% confidence interval of the repeated analysis of a given sample (if n≥4). The grey and blue bars represent the previously reported range for the modern mantle and Eoarchean mantle rocks from the Itsaq gneiss complex (33). Dark and bright shaded colors indicate the 2 s.d. uncertainty of the mean and the respective 95% confidence interval, respectively. The chromitite samples from the Itsaq gneiss complex (194856 & 194857, blue symbols) and the Bushveld igneous province (UG-2, black symbol) that were measured in this study are in accord with previous results that found anomalous and modern mantle-like μ^{100}Ru isotope compositions, respectively. Komatiites from the Dwalile greenstone remnant (AGC 83 & AGC 86, red symbols) reveal no resolvable μ^{100}Ru isotope anomalies.

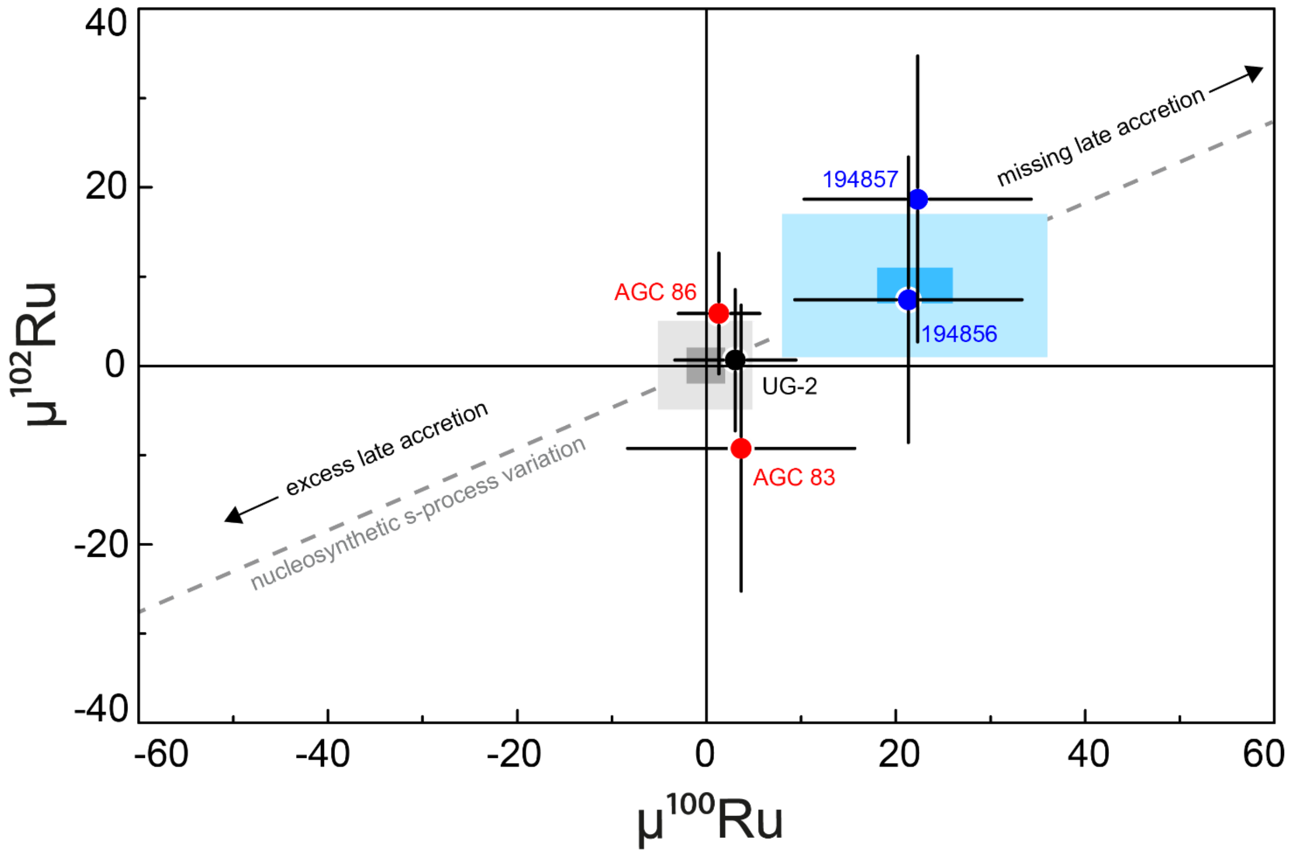

Fig. S7: Ru isotope plot illustrating the effect of heterogeneous late accretion on the Ru isotope composition in terrestrial rocks. The uncertainties for individual data points, the modern mantle (grey box) and the Eoarchean mantle in the Itsaq gneiss complex (blue box) are the same as in Fig. S6. The dashed line illustrates mixing relationships between the modern mantle composition and primitive material that has been shown to exhibit Ru isotope systematics that carry a signature of s-process nucleosynthetic composition(35). As demonstrated for Eoarchean mantle rocks from the Itsaq gneiss complex (SW Greenland) coupled $\mu^{100}Ru$-$\mu^{102}Ru$ isotope systematics in terrestrial rocks can serve as a tool to investigate to which extend mantle reservoirs equilibrated with late accreted material(33). The coupled $\mu^{100}Ru$-$\mu^{102}Ru$ isotope systematics for komatiites from the Dwalile greenstone remnant (AGC 83 & AGC 86, red symbols) overlap with the modern mantle composition and do not indicate that their mantle sources carried excess late accreted material or did not fully equilibrated with late accreted material.

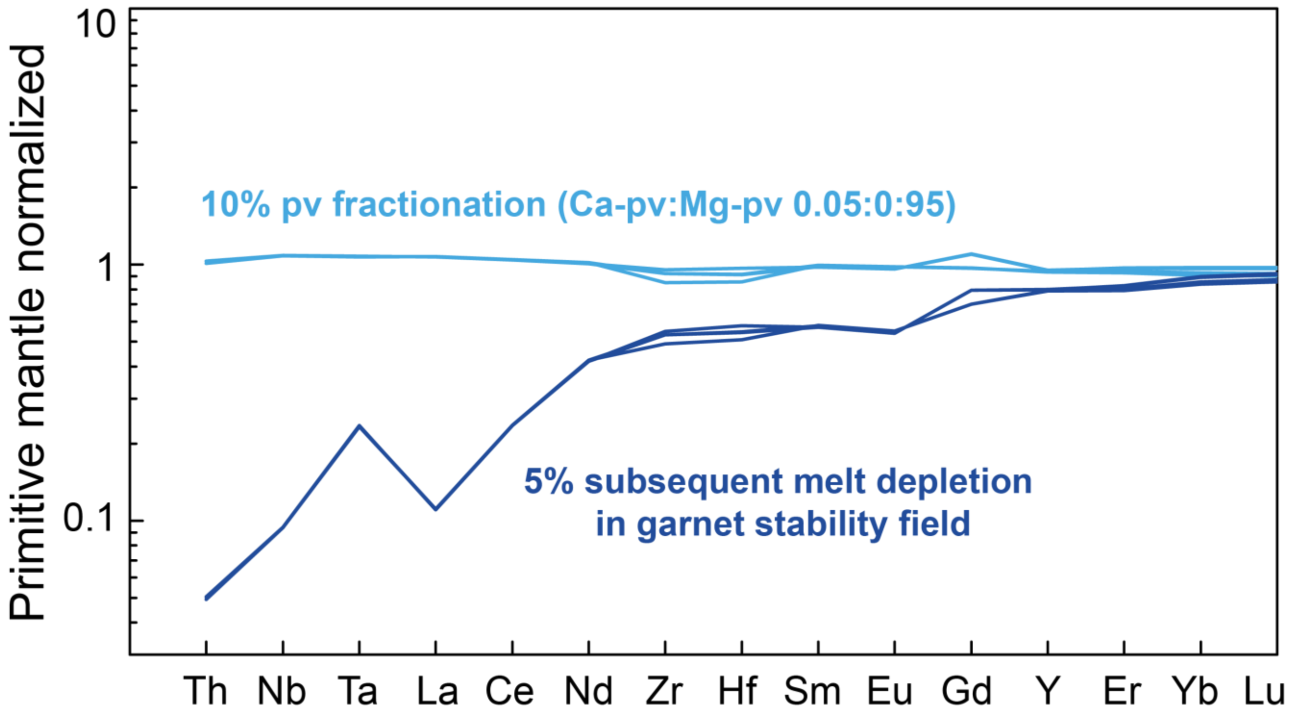

Fig. S8: Incompatible trace element compositions for primitive mantle that undergoes removal of 10% perovskite (pale blues patterns) before it undergoes batch melting in the garnet stability field (dark blue patterns). The removal of 10% perovskite does not lead to fractionated trace element patterns that are expected to explain the radiogenic initial ^{176}Hf-^{143}Nd compositions in Kaapvaal Craton rocks (Figs 3 and S12). Rather, subsequent melt depletion in the garnet stability field significantly affects the incompatible trace element budget and generates the observed ^{176}Hf-^{143}Nd systematics. Note that different lines for both reservoirs result from slight differences in perovskite partition coefficients for experimental charges(36).

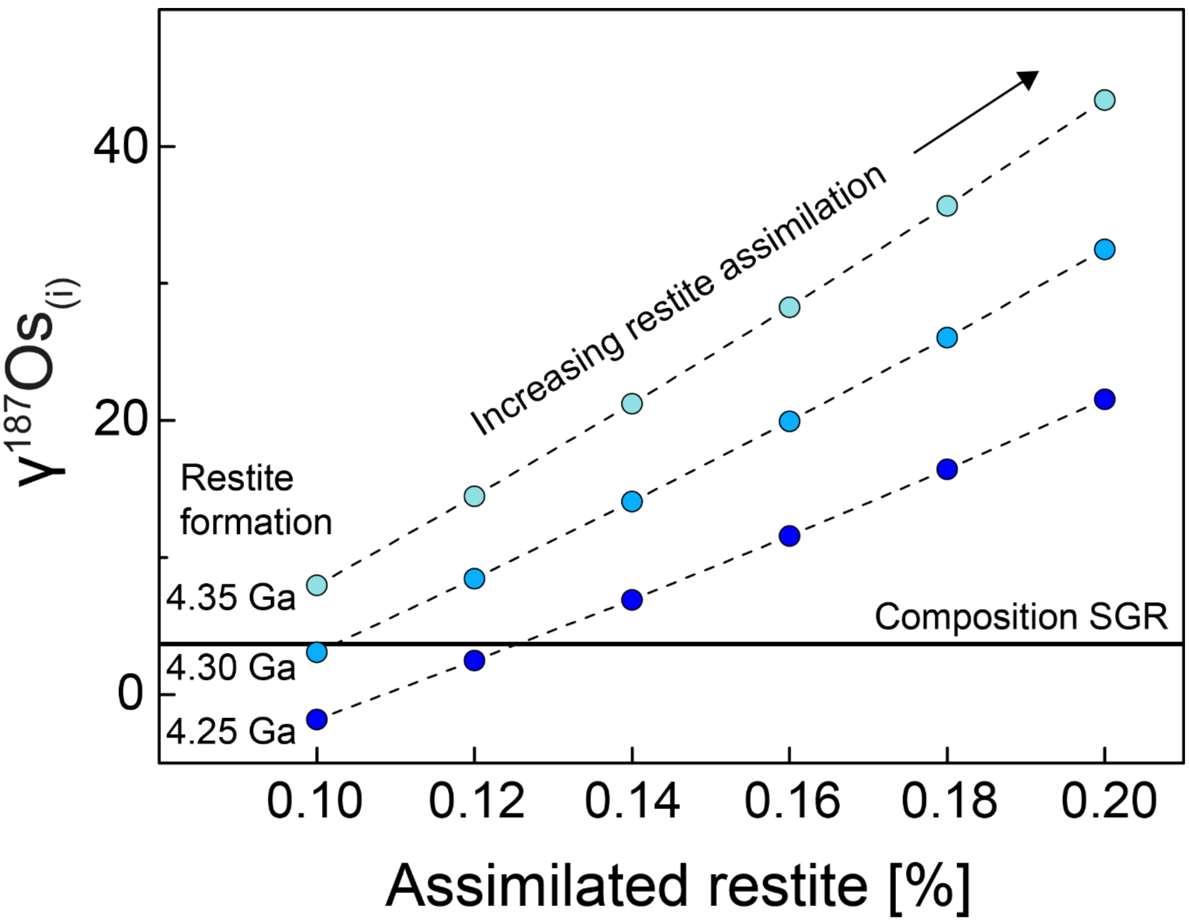

Fig. S9: Modelled $\gamma^{187}Os$ compositions for hybrid mantle reservoirs comprising depleted mantle and residual restites. Shown are modelled initial $\gamma^{187}Os$ compositions for depleted mantle that assimilated 10-20% residual restites. Following the time evolution path of our model we show mixing relationships for three generations of residual restites that formed during TTG formation between 4.35 Ga and 4.25 Ga. For explanation see method section. Calculations can be followed in Dataset S3.

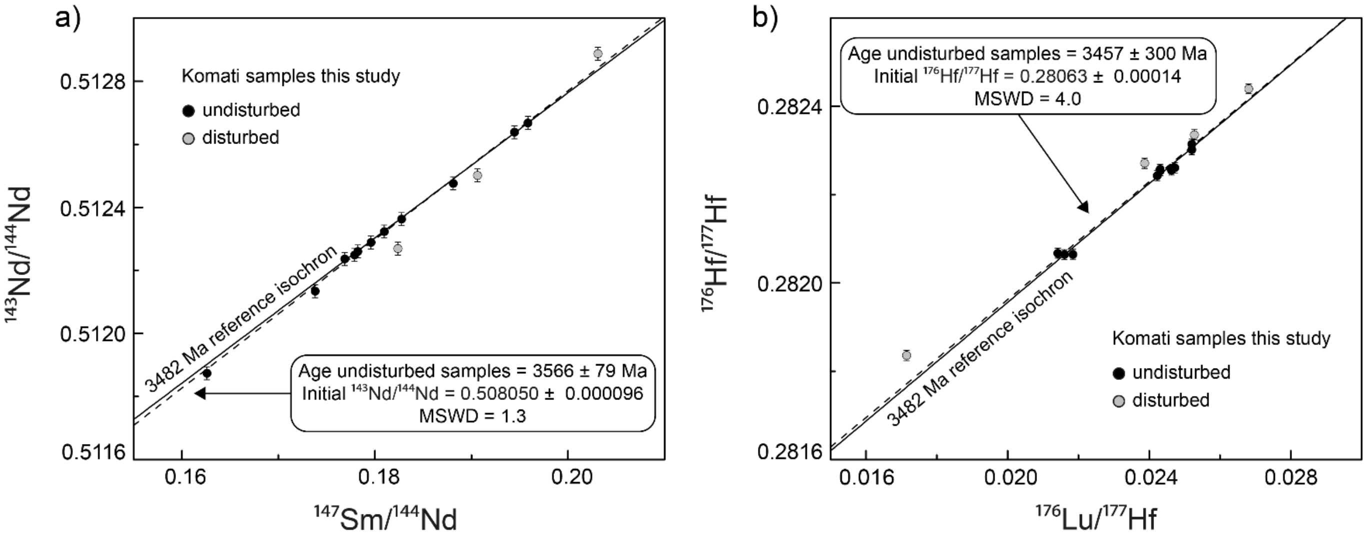

Fig. S10: Whole-rock regression lines for samples from the Komati Formation investigated in this study. Black lines represent reference isochrons for the Komati Formation that were calculated using average initial ε^{143}Nd and ε^{176}Hf values(18) and assuming an emplacement age of 3.482 Ga(47). Komati samples that do not plot along reference isochrons for ^{147}Sm-^{143}Nd (panel a) and ^{176}Lu-^{176}Hf (panel b) were regarded as being significantly disturbed (grey symbols). Excluding these samples yields age regressions of 3566 ± 76 Ma for ^{147}Sm-^{143}Nd and 3457 ± 300 Ma (dashed lines) which is in good agreement with the excepted emplacement age (3482 ± 5 Ma) obtained from single zircon U-Pb ages(47).

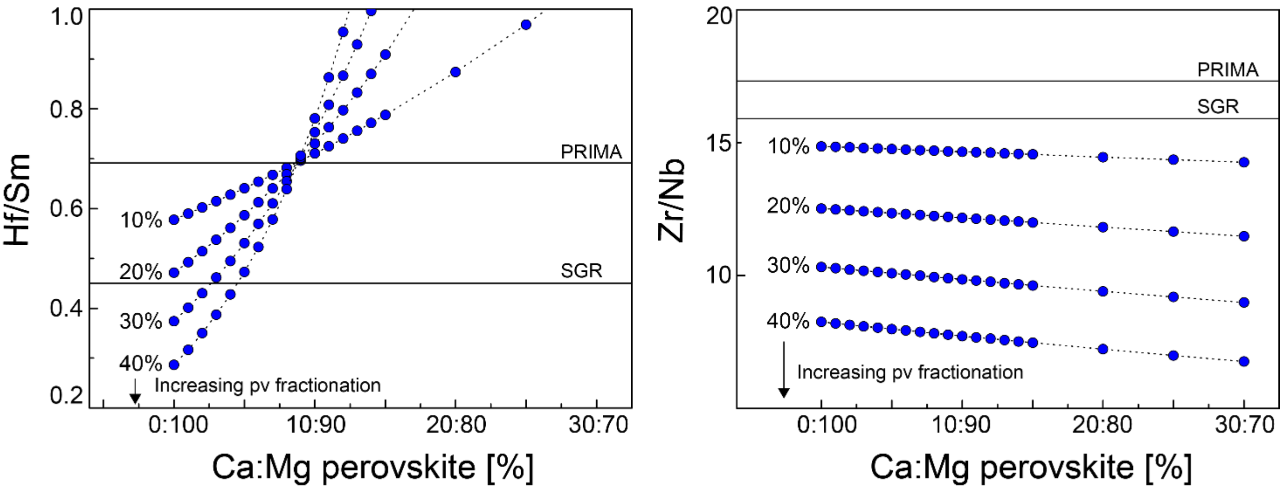

Fig. S11: Modelled effects on ratios of (a) Hf/Sm and (b) Zr/Nb in a primitive mantle like reservoir that underwent removal of different Ca-Mg perovskite assemblages, considering different Ca-Mg perovskite proportions and various degrees of perovskite fractionation. (a) The effect of Ca-Mg perovskite proportions on Hf/Sm illustrates that Ca-perovskite fractionates many trace elements in the opposite manner as Mg-perovskite, a fact that is frequently overlooked when investigating trace element ratios that behave sensitive to perovskite fractionation. **(b)** Rather, trace element ratios should be used that are largely insensitive to the choice of Ca-Mg perovskite proportions (e.g. Zr/Nb).

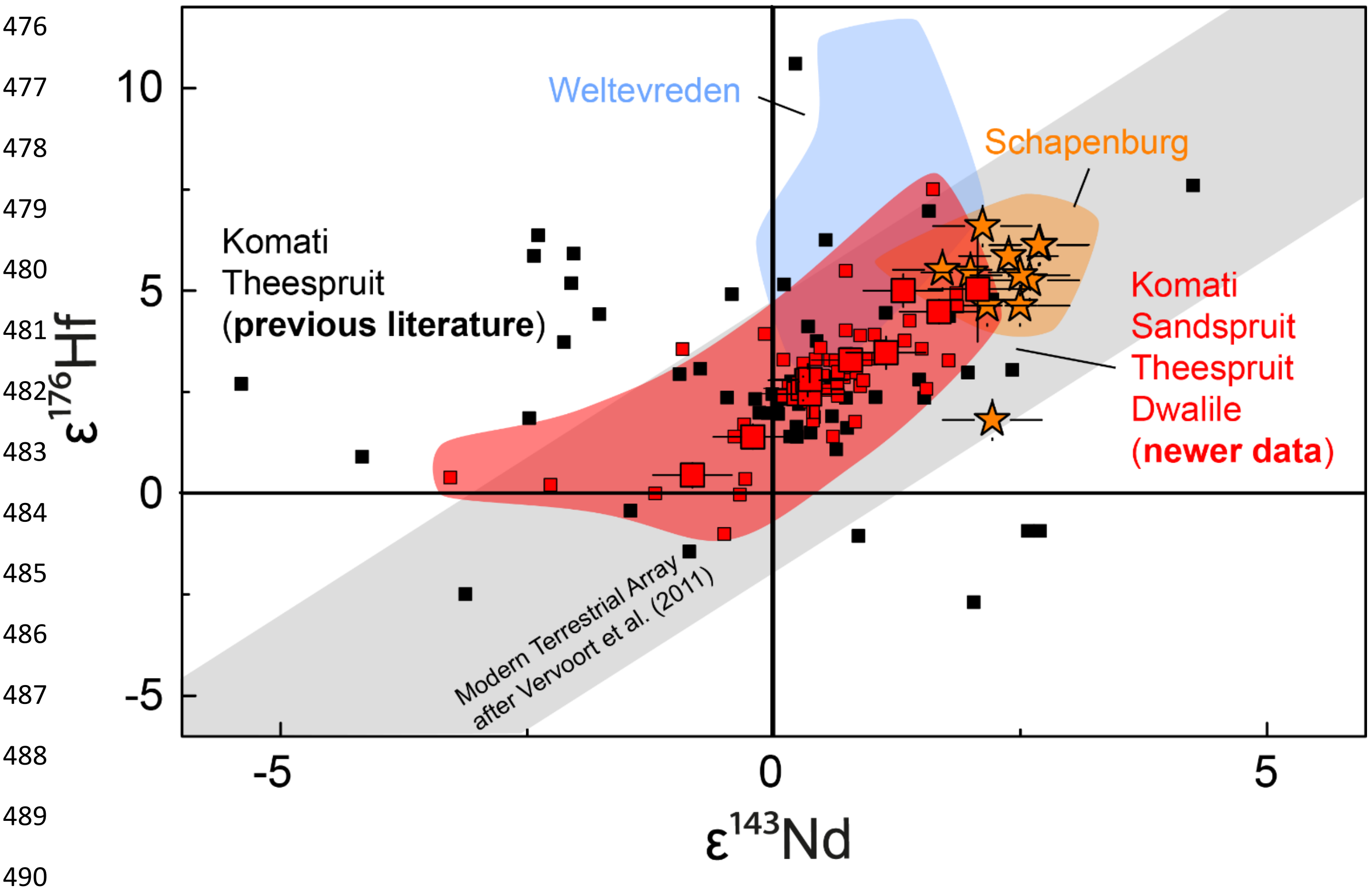

Fig. S12: $\varepsilon^{176}Hf_{(t)}$ vs. $\varepsilon^{143}Nd_{(t)}$ diagram for mantle-derived mafic-ultramafic rocks from this and previous studies. Previous literature data from the Komati and Theespruit Formations are displayed by black symbols(32, 37-40). Newer data (red symbols) are taken from more recent studies(14, 18) and are the data source for samples analyzed in this study (large red symbols). Note that newer data for the Komati formation also comprises ultramafic samples from drillcores BARB1 and BARB2 (analyzed in this study) that significantly scatter in previous datasets. The orange field is defined by komatiites from the Schapenburg Greenstone Remnant (orange stars)(30, 41, 42). The blue field shows the array for the Weltevreden komatiite suite(32, 37). The grey bar shows the Modern Terrestrial Array for MORBs and OIBs ($\varepsilon Hf = 1.55 \times \varepsilon Nd + 1.21$)(43).

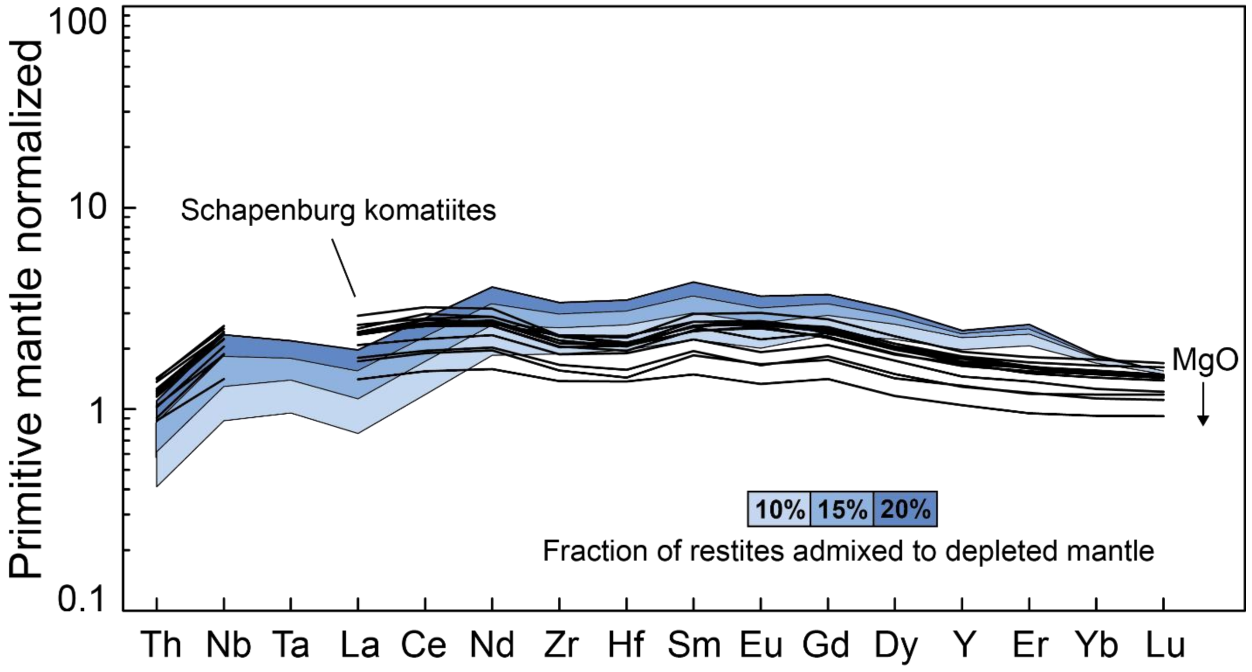

Fig. S13: Incompatibe trace element compositions for komatiites from the Schapenburg Greenstone Remnant (black lines) in comparison to melts generated from our modeled hybrid sources (blue shaded arrays). Data for the SGR komatiites are taken from the literature(30). In our model calculations 10 – 20 % of garnet – rich lower-crustal restite admixed to a depleted mantle at 3.55 Ga and subsequent 20 – 30% batch melting of this hybrid source can reproduce the trace element compositions of the SGR komatiites. As outlined previously (30,44) we attribute the variation within the SGR komatiite suite and their more depleted trace element compositions, compared to the modeled patterns, to olivine accumulation.

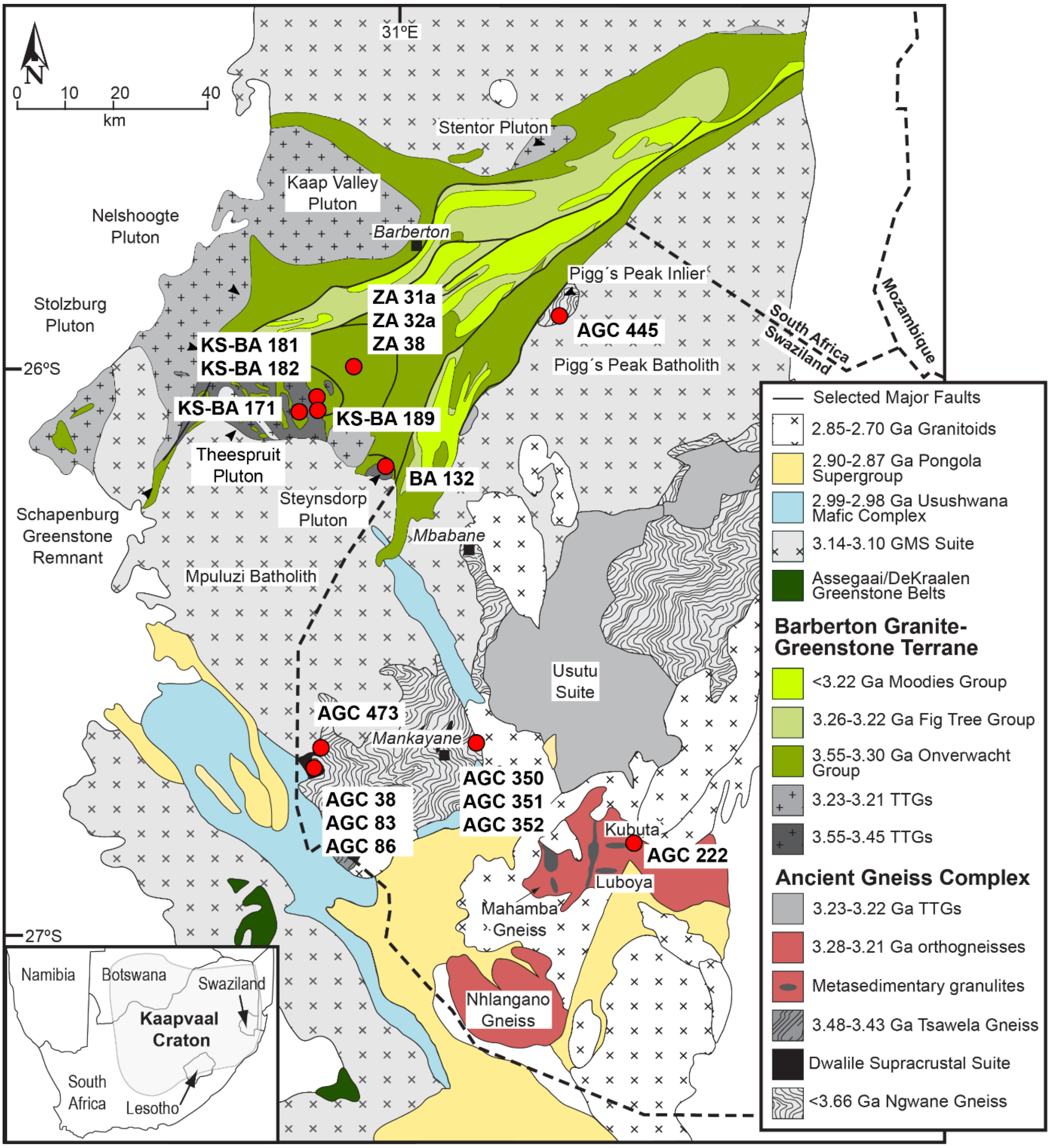

Fig. S14: Simplified geological map of the Kaapvaal Craton, Southern Africa, showing the sample localities covered in this study. The map is taken from Ref. 22 and modified after Ref. 15.

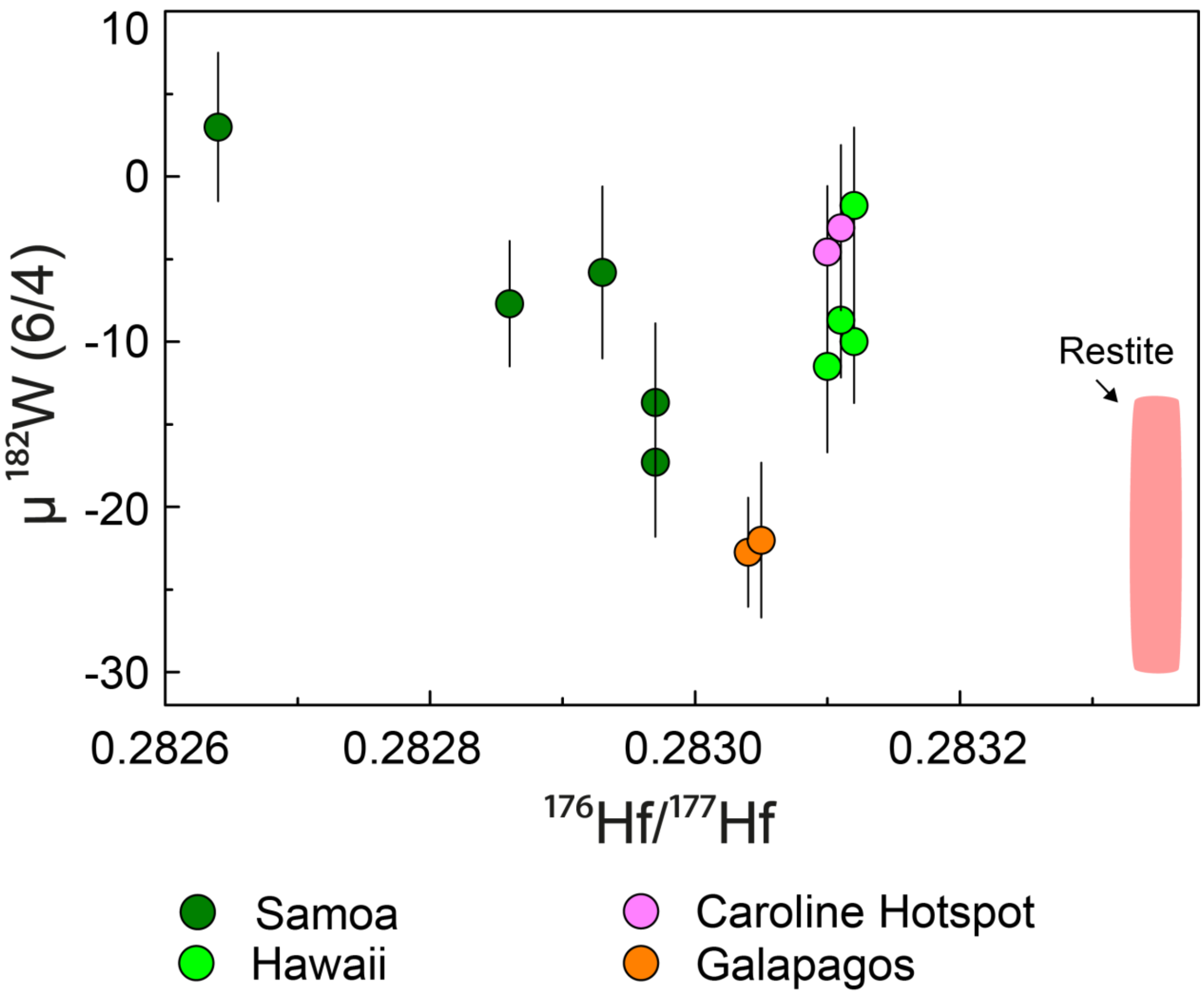

Fig. S15: Compilation of ^{182}W and ^{176}Hf isotope compositions for modern OIBs. Data were compiled from recent studies(45, 46) and references therein. As for μ^{182}W vs. ^{143}Nd/^{144}Nd (see Fig. 5) the global compilation for modern OIBs displays a similar pattern for μ^{182}W vs. ^{176}Hf/^{177}Hf, although only less constrained by the limited ^{176}Hf isotope data available. Also shown is the present ^{182}W and ^{143}Nd isotope composition calculated for restites that remained after prolonged TTG formation (4.35 – 4.25 Ga) via partial anataxis of a mafic protocrust that formed between 40 and 50 Ma after solar system formation (pink array).

References SI Appendix

1. J. E. Hoffmann, A. Kröner, “Early Archean crustal evolution in southern Africa - an updated record of the Ancient Gneiss Complex of Swaziland” in *Earth´s Oldest Rocks*, 2nd Ed., M. J. Van Kranendonk, Ed. (Elsevier, 2019), pp. 553–567.
2. A. Kröner, *et al.*, Generation of early Archaean grey gneisses through melting of older crust in the eastern Kaapvaal craton, southern Africa. *Precambrian Res.* **255**, 823–846 (2014).
3. A. Kröner, W. Compston, I. S. Williams, Growth of early Archaean crust in the Ancient Gneiss Complex of Swaziland as revealed by single zircon dating. *Tectonophysics* **161**, 271–298 (1989).
4. A. C. Wilson, 1:250,000 Geological Map of Swaziland. *Geol. Surv. Mines Dep. Mbabane, Swazil.* (1982).
5. W. Compston, A. Kröner, Multiple zircon growth within early Archaean tonalitic gneiss from the Ancient Gneiss Complex, Swaziland. *Earth Planet. Sci. Lett.* **87**, 13–28 (1988).
6. A. Zeh, A. Gerdes, L. Millonig, Hafnium isotope record of the Ancient Gneiss Complex , Swaziland , southern Africa : evidence for Archaean crust – mantle formation and crust reworking. *J. Geol. Soc. London.* **168**, 953–963 (2011).
7. B. Schoene, M. J. de Wit, S. A. Bowring, Mesoarchean assembly and stabilization of the eastern Kaapvaal craton: A structural-thermochronological perspective. *Tectonics* **27**, 1–27 (2008).
8. A. Kröner, J. I. Wendt, C. Milisenda, W. Compston, R. Maphalala, “Zircon geochronology and Nd isotopic systematics of the Ancient Gneiss Complex, Swaziland, and implications for crustal evolution” in *The Ancient Gneiss Complex: Overview Papers and Guidebook for Excursion*, Bulletin 1, A. Kröner, Ed. (Swaziland Geological Survey and Mines Department, 1993), pp. 15–37.
9. A. Kröner, Chapter 5.2 The Ancient Gneiss Complex of Swaziland and Environs: Record of Early Archean Crustal Evolution in Southern Africa. *Dev. Precambrian Geol.* **15**, 465–480 (2007).
10. M. P. A. Jackson, “Archean structural styles in the Ancient Gneiss Complex of Swaziland, South Africa” in *Precambrian Tectonics Illustrated*, A. Kröner, R. Greiling, Eds. (Schweizerbart´sche Verlagsbuchhandlung, 1984), pp. 1–18.
11. A. Kröner, A. Tegtmeyer, Gneiss-greenstone relationships in the Ancient Gneiss Complex of southwestern Swaziland, southern Africa, and implications for early crustal evolution. *Precambrian Res.* **67**, 109–139 (1994).
12. D. R. Hunter, F. Barker, H. T. Millard, Geochemical investigation of Archaean Bimodal and Dwalile metamorphic suites, Ancient Gneiss Complex, Swaziland. *Precambrian Res.* **24**, 131–155 (1984).
13. A. Kröner, *et al.*, “Archaean Crystalline Rocks of the Eastern Kaapvaal Craton” in *The Archaean Geology of the Kaapvaal Craton, Southern Africa, Regional Geology Reviews*, A. Kröner, A. Hofmann, Eds. (Springer Nature Switzerland AG, 2019), pp. 1–32.

14. J. E. Hoffmann, *et al.*, Hafnium-Neodymium isotope, trace element and U-Pb zircon age constraints on the petrogenesis of the 3.44-3.46 Ga Dwalile greenstone remnant, Ancient Gneiss Complex, Swaziland. *Precambrian Res.* **351** (2020).

15. V. van Schijndel, G. Stevens, A. Zeh, D. Frei, C. Lana, Zircon geochronology and Hf isotopes of the Dwalile Supracrustal Suite, Ancient Gneiss Complex, Swaziland: Insights into the diversity of Palaeoarchaean source rocks, depositional and metamorphic ages. *Precambrian Res.* **295**, 48–66 (2017).

16. J. E. Hoffmann, *et al.*, Source composition, fractional crystallization and magma mixing processes in the 3.48-3.43 Ga Tsawela tonalite suite (Ancient Gneiss Complex, Swaziland) - Implications for Palaeoarchaean geodynamics. *Precambrian Res.* **276**, 43–66 (2016).

17. S. B. Mukasa, A. H. Wilson, K. R. Young, Geochronological constraints on the magmatic and tectonic development of the Pongola Supergroup (Central Region), South Africa. *Precambrian Res.* **224**, 268–286 (2013).

18. K. P. Schneider, *et al.*, Petrogenetic evolution of metabasalts and metakomatiites of the lower Onverwacht Group , Barberton Greenstone Belt (South Africa). *Chem. Geol.* **511**, 152–177 (2019).

19. A. Kröner, *et al.*, High-temperature metamorphism and crustal melting at ca. 3.2 Ga in the eastern Kaapvaal craton, southern Africa. *Precambrian Res.* **317**, 101–116 (2018).

20. J. F. Moyen, G. Stevens, A. F. M. Kisters, R. W. Belcher, B. Lemirre, “TTG plutons of the Barberton granitoid-greenstone terrain, southern Africa” in *Earth´s Oldest Rocks*, 2nd Ed., M. J. Van Kranendonk, V. C. Bennett, J. E. Hoffmann, Eds. (Elsevier, 2018), pp. 615–653.

21. N. Suhr, J. E. Hoffmann, A. Kröner, S. Schröder, Archaean granulite-facies paragneisses from central Swaziland: Inferences on Palaeoarchaean crustal reworking and a complex metamorphic history. *J. Geol. Soc. London.* **172**, 139–152 (2014).

22. K. P. Schneider, J. E. Hoffmann, M. Boyet, C. Münker, A. Kröner, Coexistence of enriched and modern-like142Nd signatures in Archean igneous rocks of the eastern Kaapvaal Craton, southern Africa. *Earth Planet. Sci. Lett.* **487**, 54–66 (2018).

23. G. R. Byerly, D. R. Lowe, C. Heubeck, “Geologic evolution of the Barberton Greenstone Belt - A unique record of crustal development, surface processes, and early life 3.55 - 3.20 Ga” in *Earth´s Oldest Rocks*, 2nd Ed., M. J. Van Kranendonk, V. C. Bennett, J. E. Hoffmann, Eds. (Elsevier, 2018), pp. 569–613.

24. R. A. Armstrong, W. Compston, M. J. de Wit, I. S. Williams, The stratigraphy of the 3.5-3.2 Ga Barberton Greenstone Belt revisited: a single zircon ion microprobe study. *Earth Planet. Sci. Lett.* **101**, 90–106 (1990).

25. A. Kröner, *et al.*, Chronology of the oldest supracrustal sequences in the Palaeoarchaean Barberton Greenstone Belt, South Africa and Swaziland. *Precambrian Res.* **279**, 123–143 (2016).

26. J. C. Dann, The 3.5 Ga Komati Formation, Barberton Greenstone Belt, South Africa, Part I: New maps and magmatic architecture. *South African J. Geol.* **103**, 47–68 (2000).

27. C. R. Anhaeusser, Magmatic and structural characteristics of the ca. 3440 ma theespruit pluton, barberton mountain land, South Africa. *Am. J. Sci.* **310**, 1136–1167 (2010).

28. M. J. Van Kranendonk, A. Kröner, J. E. Hoffman, T. Nagel, C. R. Anhaeusser, Just another drip: Re-analysis of a proposed mesoarchean suture from the Barberton mountain land, South Africa. *Precambrian Res.* **254**, 19–35 (2014).

29. N. T. Arndt, *et al.*, Scientific drilling in the Barberton Greenstone Belt. *Geobulletin* **53**, 17–18 (2010).

30. Puchtel, J. Blichert-Toft, M. Touboul, M. F. Horan, R. J. Walker, The coupled 182W-142Nd record of early terrestrial mantle differentiation. *Geochemistry Geophys. Geosystems* **17**, 2168–2193 (2016).

31. M. Touboul, I. S. Puchtel, R. J. Walker, 182W evidence for long-term preservation of early mantle differentiation products. *Science (80-.).* **335**, 1065–1069 (2012).

32. I. S. Puchtel, *et al.*, Insights into early Earth from Barberton komatiites: Evidence from lithophile isotope and trace element systematics. *Geochim. Cosmochim. Acta* **108**, 63–90 (2013).

33. M. Fischer-Gödde, *et al.*, Ruthenium isotope vestige of Earth's pre-late-veneer mantle preserved in Archaean rocks. *Nature* **579**, 240–244 (2020).

34. G. J. Archer, A. Mundl, R. J. Walker, E. A. Worsham, K. R. Bermingham, High-precision analysis of 182W/184W and 183W/184W by negative thermal ionization mass spectrometry: Per-integration oxide corrections using measured 18O/16O. *Int. J. Mass Spectrom.* **414**, 80–86 (2017).

35. M. R. Savina, *et al.*, Extinct Technetium in Silicon Carbide Stardust Grains: Implications for Stellar Nucleosynthesis. *Science (80-.).* **303**, 649–652 (2004).

36. A. Corgne, C. Liebske, B. J. Wood, D. C. Rubie, D. J. Frost, Silicate perovskite-melt partitioning of trace elements and geochemical signature of a deep perovskitic reservoir. *Geochim. Cosmochim. Acta* **69**, 485–496 (2005).

37. J. Blichert-Toft, N. T. Arndt, A. Wilson, G. Coetzee, Hf and Nd isotope systematics of early Archean komatiites from surface sampling and ICDP drilling in the Barberton Greenstone Belt, South Africa. *Am. Mineral.* **100**, 2396–2411 (2015).

38. J. Blichert-Toft, N. T. Arndt, Hf isotope compositions of komatiites. *Earth Planet. Sci. Lett.* **171**, 439–451 (1999).

39. Y. Lahaye, *et al.*, The influence of alteration on the trace-element and Nd isotopic compositions of komatiites. *Chem. Geol.* **126**, 43–64 (1995).

40. A. Kröner, *et al.*, Generation of early Archaean felsic greenstone volcanic rocks through crustal melting in the Kaapvaal, craton, southern Africa. *Earth Planet. Sci. Lett.* **381**, 188–197 (2013).

41. J. Blichert-Toft, N. T. Arndt, G. Gruau, Hf isotopic measurements on Barberton

komatiites: Effects of incomplete sample dissolution and importance for primary and secondary magmatic signatures. *Chem. Geol.* **207**, 261–275 (2004).

42. C. Lécuyer, G. gruau, C. R. Anhaeusser, S. Fourcade, The origin of fluids and the effects of metamorphism on the primary chemical compositions of Barberton komatiites: New evidence from geochemical (REE) and isotopic (Nd, O, H, 39Ar40Ar) data. *Geochim. Cosmochim. Acta* **58**, 969–984 (1994).

43. J. D. Vervoort, T. Plank, J. Prytulak, The Hf-Nd isotopic composition of marine sediments. *Geochim. Cosmochim. Acta* **75**, 5903–5926 (2011).

44. I. S. Puchtel, R. J. Walker, C. R. Anhaeusser, G. Gruau, Re-Os isotope systematics and HSE abundances of the 3.5 Ga Schapenburg komatiites, South Africa: Hydrous melting or prolonged survival of primordial heterogeneities in the mantle? *Chem. Geol.* **262**, 355–369 (2009).

45. A. Mundl-Petermeier, *et al.*, Anomalous 182W in high 3He/4He ocean island basalts: Fingerprints of Earth's core? *Geochim. Cosmochim. Acta* **271**, 194–211 (2020).

46. M. G. Jackson, J. Blichert-toft, S. A. Halldórsson, A. Mundl-petermeier, Ancient helium and tungsten isotopic signatures preserved in mantle domains least modified by crustal recycling. *Proc. Natl. Acad. Sci.* **117**, 30993–31001 (2020).

47. R. A. Armstrong, W. Compston, M. J. de Wit, I. S. Williams, The stratigraphy of the 3.5-3.2 Ga Barberton Greenstone Belt revisited: a single zircon ion microprobe study. *Earth Planet. Sci. Lett.* **101**, 90–106 (1990).
